# A Putative Bacterial ABC Transporter Circumvents the Essentiality of Signal Peptidase

**DOI:** 10.1128/mBio.00412-16

**Published:** 2016-09-06

**Authors:** J. Hiroshi Morisaki, Peter A. Smith, Shailesh V. Date, Kimberly K. Kajihara, Chau Linda Truong, Zora Modrusan, Donghong Yan, Jing Kang, Min Xu, Ishita M. Shah, Robert Mintzer, Eric M. Kofoed, Tommy K. Cheung, David Arnott, Michael F. T. Koehler, Christopher E. Heise, Eric J. Brown, Man-Wah Tan, Wouter L. W. Hazenbos

**Affiliations:** a Department of Infectious Diseases, Genentech, Inc., South San Francisco, California, USA; b Department of Molecular Biology, Genentech, Inc., South San Francisco, California, USA; c Department of Translational Immunology, Genentech, Inc., South San Francisco, California, USA; d Department of Biochemical and Cellular Pharmacology, Genentech, Inc., South San Francisco, California, USA; e Department of Protein Chemistry, Genentech, Inc., South San Francisco, California, USA; f Department of Discovery Chemistry, Genentech, Inc., South San Francisco, California, USA

## Abstract

The type I signal peptidase of *Staphylococcus aureus*, SpsB, is an attractive antibacterial target because it is essential for viability and extracellularly accessible. We synthesized compound 103, a novel arylomycin-derived inhibitor of SpsB with significant potency against various clinical *S. aureus* strains (MIC of ~1 µg/ml). The predominant clinical strain USA300 developed spontaneous resistance to compound 103 with high frequency, resulting from single point mutations inside or immediately upstream of *cro*/*cI*, a homolog of the lambda phage transcriptional repressor *cro*. These *cro*/*cI* mutations led to marked (>50-fold) overexpression of three genes encoding a putative ABC transporter. Overexpression of this ABC transporter was both necessary and sufficient for resistance and, notably, circumvented the essentiality of SpsB during *in vitro* culture. Mutation of its predicted ATPase gene abolished resistance, suggesting a possible role for active transport; in these bacteria, resistance to compound 103 occurred with low frequency and through mutations in *spsB*. Bacteria overexpressing the ABC transporter and lacking SpsB were capable of secreting a subset of proteins that are normally cleaved by SpsB and instead were cleaved at a site distinct from the canonical signal peptide. These bacteria secreted reduced levels of virulence-associated proteins and were unable to establish infection in mice. This study reveals the mechanism of resistance to a novel arylomycin derivative and demonstrates that the nominal essentiality of the *S. aureus* signal peptidase can be circumvented by the upregulation of a putative ABC transporter *in vitro* but not *in vivo*.

## IMPORTANCE

The type I signal peptidase of *Staphylococcus aureus* (SpsB) enables the secretion of numerous proteins by cleavage of the signal peptide. We synthesized an SpsB inhibitor with potent activity against various clinical *S. aureus* strains. The predominant *S. aureus* strain USA300 develops resistance to this inhibitor by mutations in a novel transcriptional repressor (*cro*/*cI*), causing overexpression of a putative ABC transporter. This mechanism promotes the cleavage and secretion of various proteins independently of SpsB and compensates for the requirement of SpsB for viability *in vitro*. However, bacteria overexpressing the ABC transporter and lacking SpsB secrete reduced levels of virulence-associated proteins and are unable to infect mice. This study describes a bacterial resistance mechanism that provides novel insights into the biology of bacterial secretion.

## INTRODUCTION

Discovery of novel antibiotics has become an important goal for biomedical research in both academia and industry, mainly driven by the growing problem of widespread antibiotic resistance (1). *Staphylococcus aureus* is a major threat to human health and can cause life-threatening invasive infections, such as bacteremia, endocarditis, pneumonia, and osteomyelitis (2). Infections with *S. aureus* have become increasingly difficult to treat because of the emergence of methicillin resistance and high failure rates of standard-of-care antibiotics like vancomycin (3).

The number of suitable antibiotic targets in *S. aureus* is rather limited compared to the number of genes found to be essential in genetic screens (4), largely because of the difficulty of finding cell-active inhibitors for essential enzymes that can penetrate the bacterial cell wall and avoid efflux mechanisms. The type I signal peptidase of *S. aureus*, SpsB, is an attractive antibiotic target because it is essential for viability and accessible on the extracellular side of the bacterial membrane, while its active site is different from that of human signal peptidases (5). SpsB substrates are membrane-bound preproteins secreted by the Sec secretion pathway that contain a canonical Ala-X-Ala motif in the signal sequence (6, 7). Inhibition of SpsB activity leads to a loss of bacterial viability (8, 9).

A recently discovered natural compound, arylomycin (10, 11), has been reported to inhibit type I signal peptidases of various bacterial species by occupying the enzymatic groove that accommodates natural substrates during the process of secretion (12). However, the potency of arylomycin against *S. aureus* is too low to be clinically useful for treatment of *S. aureus* infections (5, 13).

This study describes a novel SpsB inhibitor, the arylomycin analog compound 103, which has enhanced potency against *S. aureus*. We found that resistance to this inhibitor occurs with high frequency *in vitro* and is mediated by robust overexpression of a putative ABC transporter that results from loss-of-function mutations in *cro*/*cI*, a homolog of the lambda phage transcriptional repressor *cro* gene. The overexpression of this ABC transporter prevented bacterial lethality caused by disruption of the *spsB* gene. This was associated with secretion of a subset of proteins that are normally cleaved by SpsB and were cleaved at a site distinct from the canonical SpsB cleavage site. Bacteria reliant on this secretion mechanism secreted reduced levels of functional virulence-associated proteins and were unable to infect mice, demonstrating an absolute requirement for SpsB activity during infection. This study reveals a novel bacterial resistance mechanism that led to the discovery of an alternative system for cleavage and secretion of signal peptide-containing proteins that counteracts the essentiality of SpsB *in vitro* but not *in vivo*.

## RESULTS AND DISCUSSION

### Anti-*S. aureus* activity of SpsB inhibitor compound 103.

Arylomycins are a naturally occurring family of structurally related antibiotics that inhibit SpsB of *S. aureus*, albeit with low antibacterial potency (5, 13). In an effort to create novel antibiotics to treat *S. aureus* infections, we synthesized compound 103 (see Fig. S1A in the supplemental material), a new analog of arylomycin (see Fig. S1B) with improved activity against *S. aureus*. The MIC of compound 103 for the predominant clinical methicillin-resistant *S. aureus* (MRSA) strain USA300 was 1.0 µg/ml, whereas the MIC for arylomycin A_16_ was 32 µg/ml (this study). Compound 103 exhibited MICs ranging from 0.5 to 1.4 µg/ml for a panel of eight clinical *S. aureus* strains (Table 1). Compound 103 dose dependently inhibited the enzymatic activity of recombinant SpsB (see Fig. S1C). The antibacterial activity of compound 103 occurred specifically through SpsB, as confirmed by a reduced MIC for a USA300 strain that underexpresses SpsB and an increased MIC for an SpsB-overexpressing strain (Table 1).

**TABLE 1:** MICs of compound 103 for *S. aureus* strains

*S. aureus* strain	Resistance type^a^	MIC of compound 103 (μg/ml)
USA300 wild type	MRSA	1.0
USA300 SpsB-high	MRSA	64
USA300 SpsB-low	MRSA	0.125
COL	MRSA	0.7
MRSA252	MRSA	1.0
Mu50	VISA	1.0
USA100	MRSA	0.5
USA1000	MRSA	1.0
USA400	MRSA	1.4
Newman	MSSA	1.0

a MRSA, methicillin-resistant *S. aureus*; MSSA, methicillin-sensitive *S. aureus*; VISA, vancomycin-intermediate *S. aureus*.

### Resistance to compound 103 is caused by mutations in *SAUSA300_0350* (*cro*/*cI*).

To determine the mechanism of spontaneous resistance against compound 103, we selected 40 independently generated resistant mutants of *S. aureus* USA300, which arose at a frequency of 3 × 10^−7^ from cultures on agar containing compound 103 at fourfold its MIC. The MICs of compound 103 for all of these mutants were increased by at least 16-fold compared to the MIC of wild-type (WT) USA300.

**FIG 1:**
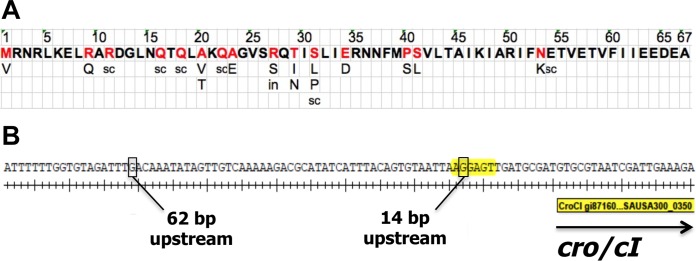
*cro*/*cI* mutations that are associated with resistance to SpsB inhibitor compound 103. (A) Predicted full-length amino acid sequence of Cro/CI protein. Amino acids that were found to be mutated in resistant clones are indicated in red, with their respective substitutions underneath. sc, stop codon; in, insertion of CGTTTCAAGCGGG resulting in multiple stop codons. (B) Single-base-pair substitutions that were found in the promoter region of the *cro*/*cI* open reading frame (ORF) and that were associated with resistance to compound 103 were located 62 bp and 14 bp upstream of the *cro*/*cI* ORF. All mutations inside (A) or upstream of (B) the *cro*/*cI* ORF are associated with a >16-fold increase in the MIC of compound 103.

Whole-genome sequencing of all 40 mutants revealed that resistance was associated with a single mutation inside or just upstream of gene *SAUSA300_0350* in all of these clones. Based on homology to the lambda phage Cro protein, *SAUSA300_0350* is annotated as “Cro/CI transcriptional regulator-like protein” and will be referred to as *cro*/*cI* throughout this paper. We identified mutations in 16 of the 67 amino acids of the predicted Cro/CI protein (Fig. 1A; see also Table S1 in the supplemental material), including multiple substitutions or stop codons and one insertion. We also identified two single-nucleotide substitutions immediately upstream of the *cro*/*cI* translational start associated with resistance (Fig. 1B), i.e., a change of G to T 14 bp upstream of *cro/cI*, located in a predicted ribosomal binding site (RBS) (AGGAGT) in the *cro*/*cI* promoter and likely leading to defective Cro/CI protein translation, and a change of G to A 62 bp upstream of *cro*/*cI*.

Plasmid-encoded expression of exogenous WT *cro*/*cI* reversed the resistant phenotype of clones with mutations inside *cro*/*cI* or at 14 bp upstream of *cro*/*cI* to a sensitive phenotype (Table 2, strains GNE0109 and GNE0097). This confirms that loss-of-function mutations in *cro*/*cI* are the cause of resistance to compound 103. WT *cro*/*cI* could not complement the phenotype of the G-to-A mutation 62 bp upstream of *cro*/*cI* (Table 2, strain GNE0100), indicating that this mutation is presumably located in the putative Cro/CI binding site, by analogy to the lambda phage *cro* gene (14, 15). Mutation of *cro*/*cI* did not reduce sensitivity to other tested antibiotics, including vancomycin, linezolid, oxacillin, daptomycin, rifampin, gentamicin, clindamycin, erythromycin, gatifloxacin, and novobiocin (see Table S2 in the supplemental material). The loss-of-function *cro*/*cI* mutation did not result in a significant alteration in virulence in a mouse infection model (see Fig. S2).

**TABLE 2:** Roles of *cro*/*cI* and putative ABC transporter genes in resistance of USA300-derived strains to compound 103

Strain	Relevant genotype^a^	MIC of compound 103 (fold increase versus MIC for USA300 WT)	Expression of *SAUSA300_0352* (fold increase versus level in USA300 WT)
GNE0163	USA300 WT(pMK4)	1.0	1.0
GNE0162	p*sarA-SAUSA300*_*0351*_*0352*_*0353*	>32	56.9
GNE0107	*cro*/*cI*(M1V)(pMK4)	>32	66.2
GNE0109	*cro*/*cI*(M1V)(p*cro*/*cI*-*cro*/*cI*)	1.7	1.0
GNE0096	*cro*/*cI*(−14G→T)(pMK4)	>32	10.9
GNE0097	*cro*/*cI*(−14G→T)(p*cro*/*cI*-*cro*/*cI*)	3.0	0.1
GNE0099	*cro*/*cI*(−62G→A)(pMK4)	>32	5.5
GNE0100	*cro*/*cI*(−62G→A)(p*cro/cI*-*cro*/*cI*)	>32	8.0
GNE0173	Δ*mcr cro*/*cI*(Q16stop)	>32	105
GNE0220	Δ*mcr cro*/*cI*(Q16stop) Δ(*SAUSA300_0351-SAUSA300*_*0352*-*SAUSA300*_*0353*)	2.0	ND^b^
GNE0209	Δ*mcr cro*/*cI*(Q16stop) Δ*SAUSA300*_*0351*	>32	93
GNE0175	Δ*mcr cro*/*cI*(Q16stop) *SAUSA300_0352*(K44A)	1.0	106
GNE0210	Δ*mcr cro*/*cI*(Q16stop) Δ*SAUSA300*_*0353*	1.0	ND

a All strains are USA300 derived, and all plasmids are pMK4 (empty plasmid) or constructed in pMK4. A more detailed description of each strain is given in Table S5 in the supplemental material. *SAUSA300*_*0351* encodes a putative membrane protein, *SAUSA300_0352* encodes a putative ABC transporter ATPase, and *SAUSA300*_*0353* encodes a putative permease.

b ND, not determined.

A recent study suggested a Gln at position 22 (Gln22) in a *cro*/*cI* homolog in *S. aureus* strain N315 to be associated with resistance to M131, another arylomycin derivative (16). According to the public NCBI database, Gln22 is present in the sequences of several *S. aureus* strains that are sensitive to compound 103, such as COL, Mu50, and MRSA252, as well as the USA300 strain we used in the present study (Table 1). Therefore, a Gln at position 22 in *cro*/*cI* does not confer resistance to compound 103. Instead, we did observe that the replacement of Gln22 with a stop codon (Q22stop) was associated with resistance to compound 103 (Fig. 1A).

We next investigated whether *S. aureus* *cro/cI* mutations preexist in nature. Among a panel of 102 globally diverse clinical *S. aureus* isolates, 7 isolates showed resistance to compound 103, each exhibiting a MIC of >32 µg/ml. Compared with the corresponding sequences from 5 compound 103-sensitive strains (strains USA300, COL, MRSA252, USA400, Mu50) (Table 1), all 7 resistant isolates showed an AG-to-TA nucleotide substitution at 67/68 bp upstream of *cro*/*cI*, while their *spsB* sequences were identical to the *spsB* sequences of these 5 sensitive strains. Analogous to the mutation we detected 62 bp upstream of *cro*/*cI* in USA300 (Fig. 1B), we speculate that the resistance of these 7 clinical isolates to compound 103 may be caused by mutation of the Cro/CI binding site in the operator region.

In summary, our comprehensive analysis identified a number of recessive mutations in *cro*/*cI* or in its predicted RBS and a mutation in a putative operator region of *cro*/*cI* that was quite similar to a naturally occurring mutation in about 7% of clinical *S. aureus* isolates tested, all of which could lead to loss of Cro/CI function. Thus, we hypothesized that loss of Cro/CI function is the most common mechanism of resistance to SpsB inhibitor compound 103.

### Cro/CI is a previously uncharacterized transcriptional regulator of *S. aureus* and suppresses the expression of a putative ABC transporter.

Lambda phage Cro regulates the expression of target genes that are located in its operon, through interaction at an upstream operator sequence (14, 15). We tested the hypothesis that the *cro*/*cI* gene product in *S. aureus* functions as a transcriptional regulator by analyzing the transcription profiles of *cro*/*cI* mutants.

**FIG 2:**
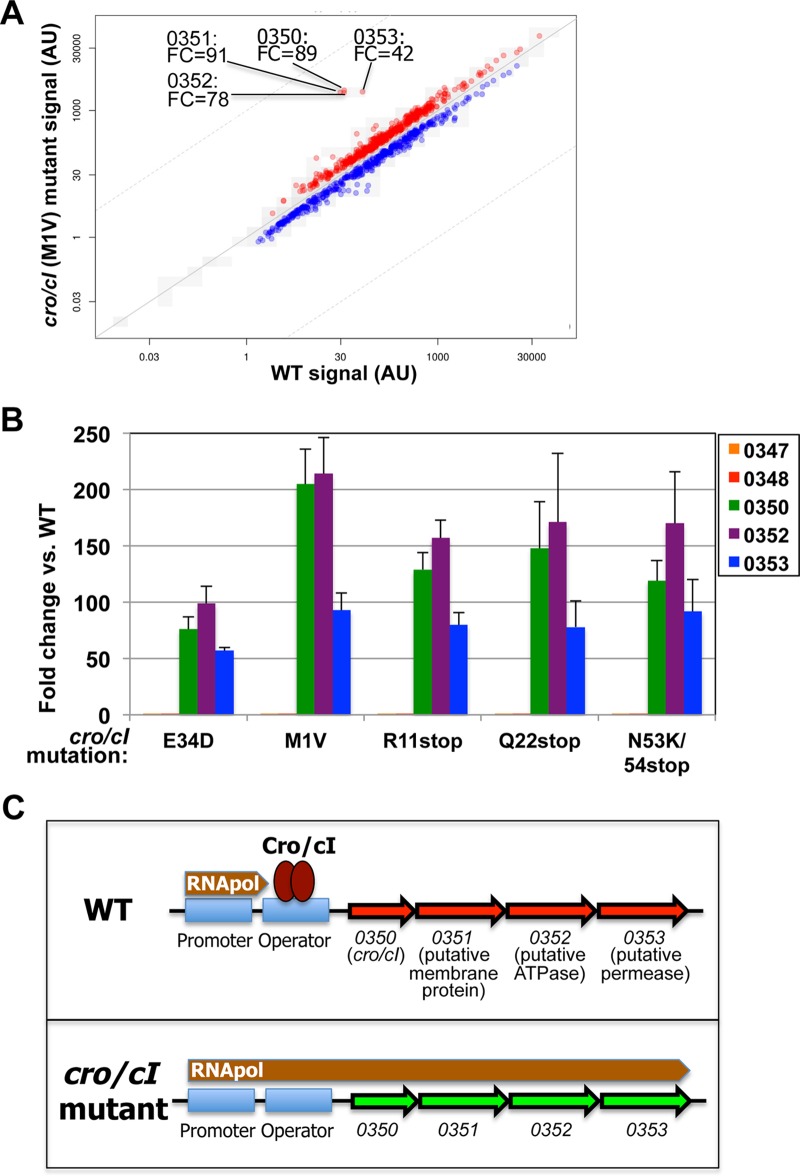
*cro*/*cI* mutations enhance the expression of all 4 genes in the *cro*/*cI*-ABC transporter operon. (A) RNA sequencing showing differential expression of 4 genes in *S. aureus* USA300 *cro*/*cI*(M1V) mutant (strain GNE0117) versus WT USA300. This revealed a significant enhancement in transcript expression of four genes sharing an operon, including *SAUSA300_0350* (*cro*/*cI*) itself (89-fold enhanced) and three genes encoding a putative ABC transporter, i.e., *SAUSA300_0351* (0351, product annotated as a putative membrane protein; 91-fold upregulated), *SAUSA300_0352* (0352, encoding a putative ATPase; 78-fold), and *SAUSA300_0353* (0353, encoding a putative permease; 42-fold). Similar results were obtained for three other *cro*/*cI* mutants, i.e., *cro*/*cI*(R11stop) (strain GNE0215), *cro*/*cI*(E34D) (strain GNE0214), and *cro*/*cI*(N53K/54stop) (strain GNE0217) mutants. FC, fold change compared to expression in WT USA300; AU, arbitrary units. Table S3 in the supplemental material shows all other genes that were upregulated by at least twofold. (B) Transcript expression of genes upstream of or inside the *cro*/*cI* operon was quantified by qRT-PCR in five different USA300 *cro*/*cI* mutants (with E34D, M1V, R11stop, Q22stop, and N53K/54stop mutations). This confirmed the robust (>50-fold) overexpression of genes located inside the *cro*/*cI* operon, i.e., *SAUSA300_0350* (*cro*/*cI*), *SAUSA300_0352*, and *SAUSA300_0353*. The expression of two genes located immediately upstream of *cro*/*cI*, i.e., *SAUSA300_0347* (annotated as *tatC*) and *SAUSA300_0348* (*tatA*), was not affected. Shown is the fold change for each mutant versus the expression in WT USA300, representing the average ± standard deviation (SD) of three independent values. (C) Hypothetical model for the role of *cro*/*cI*-mediated regulation of the putative ABC transporter in *S. aureus* resistance to compound 103. In WT *S. aureus* (top), Cro/CI protein binds to the operator sequence, suppressing transcription of the ABC transporter by blocking RNA polymerase activity, analogous to the effects of Cro of lambda phage (15). In a *cro*/*cI* mutant (bottom), either no Cro/CI protein or a nonfunctional Cro/CI protein is produced, enabling polymerase activity that leads to overexpression of the ABC transporter, which causes resistance to compound 103.

RNA sequencing of four *cro*/*cI* mutants (carrying Cro/CI mutations of M to V at position 1 [M1V] [strain GNE0117], R11stop [strain GNE0215], E34D [strain GNE0214], and N53K/54stop [strain GNE0217]) revealed that the expression levels of four genes were 42- to 91-fold greater than in the WT, while the expression levels of all other genes ranged within a 1- to 6-fold difference from their levels in wild-type bacteria (Fig. 2A; see also Table S3 in the supplemental material). These four most highly overexpressed genes are located in a predicted operon that includes the following genes: *cro*/*cI* itself; a gene encoding a putative ABC transporter protein, annotated as “putative membrane protein,” i.e., *SAUSA300_0351*; *SAUSA300_0352*, whose product is annotated as “multidrug ABC transporter ATP-binding domain”; and *SAUSA300_0353*, whose product is annotated as “putative multidrug ABC transporter permease.” Reverse transcription-quantitative PCR (qRT-PCR) analysis confirmed overexpression of the *cro*/*cI* operon genes by more than 50-fold in five different *cro*/*cI* mutants (Fig. 2B).

Exogenous expression of plasmid-encoded WT *cro*/*cI* in a *cro*/*cI*(M1V) mutant reversed the transcription of *SAUSA300_0352* to WT levels (Table 2, strain GNE0109), demonstrating that intact *cro*/*cI* actively suppresses the transcription of the putative ABC transporter. The transcription of two genes located immediately upstream of *cro*/*cI*, i.e., *SAUSA300_0347* (whose product is annotated as “TatC secretion protein”) and *SAUSA300_0348* (whose product is annotated as “TatA secretion protein”), was not affected by *cro*/*cI* mutations (Fig. 2B). These data confirm that *S. aureus* Cro/CI is a transcriptional repressor with the putative ABC transporter as its critical target.

A proposed model for the regulation of the ABC transporter by Cro/CI is shown in Fig. 2C. In WT bacteria, the expression of the putative ABC transporter genes (*SAUSA300_0351*, *_0352*, and *_0353*) is suppressed by binding of Cro/CI to the operator, located upstream of *cro*/*cI* (analogous to lambda phage Cro [14, 15]). In *cro*/*cI* mutants, either no Cro/CI protein or a nonfunctional Cro/CI protein is produced, or the affinity of the Cro/CI-binding site is reduced, in each case enabling RNA polymerase to initiate transcription of the operon genes, leading to overexpression of the ABC transporter.

### The putative ABC transporter is necessary and sufficient for high-level resistance to compound 103.

A series of genetic experiments established the role of the putative ABC transporter in resistance to compound 103. Deletion of all 3 ABC transporter genes (*SAUSA300_0351*, *_0352*, and *_0353*) reversed the resistant phenotype of a USA300 *cro*/*cI* mutant to a sensitive phenotype (Table 2, strain GNE0220). Conversely, overexpression of these 3 genes in WT USA300 induced resistance to compound 103 (Table 2, strain GNE0162). Thus, overexpression of the genes for the predicted ABC transporter is necessary and sufficient to mediate full resistance to compound 103.

Next, we determined which of these three individual genes were required for resistance. Deletion of *SAUSA300_0351* (encoding the putative membrane protein) in a resistant *cro*/*cI* mutant had no effect on the resistance phenotype (Table 2, strain GNE0209). qRT-PCR analysis confirmed the absence of transcripts of *SAUSA300_0351* in this deletion mutant (not shown), while the overexpression of *SAUSA300_0352* (Table 2) and *SAUSA300_0353* (not shown) transcripts was similar to their transcription in the parental *cro*/*cI* mutant (strain GNE0193). Substitution of Lys to Ala at position 44 (K44A) within the Walker A motif of the gene encoding the predicted ATPase, *SAUSA300_0352*, known to prevent nucleotide binding in other ABC transporters (17, 18), reversed the resistant phenotype of a *cro*/*cI* mutant to a sensitive phenotype (Table 2, strain GNE0175). This *SAUSA300_0352*(K44A) point mutant showed unaltered transcript overexpression of *SAUSA300_0352* itself (Table 2) and of *SAUSA300_0353* (not shown). Thus, high-frequency resistance to compound 103 requires ATP binding by the *SAUSA300_0352* gene product and, presumably, involves active transport by the putative ABC transporter. Deletion of *SAUSA300_0353* also reversed the resistant phenotype of the *cro*/*cI* mutant, consistent with the predicted role of its product as an ABC transporter permease (Table 2, strain GNE0210).

Inactivation of the putative ABC transporter by a K44A substitution in *SAUSA300_0352* in a WT USA300 background (generating strain GNE0174) reduced the frequency of spontaneous resistance to compound 103 by approximately 2 orders of magnitude, to 2 × 10^−9^. In the resistant mutants that arose from this strain, we identified mutations in *spsB* leading to substitutions of 4 amino acids in SpsB (Y30, V49, H68, and V167), which were each associated with an ~4- to 8-fold increased MIC for compound 103 (Table 3), presumably due to reduced affinity of SpsB for compound 103. Thus, inactivation of the putative ABC transporter eliminates the effects of *cro*/*cI* mutations, resulting in low-frequency resistance of USA300 to compound 103 that is associated with on-target mutations in *spsB*.

**TABLE 3:** SpsB point mutations found in the USA300 Δ*mcr cro*/*cI*(Q16stop) *SAUSA300_0352*(K44A) mutant^a^

Amino acid substitution	MIC of compound 103 (fold increase versus MIC for USA300 WT)
Y30C	4
Y30N	4
Y30D	8
Y30S	4
V49L	4
H68Y	8
H69L	4
V167D	8
V167F	8

a Resistant clones were selected from USA300 Δ*mcr cro*/*cI*(Q16stop) *SAUSA300_0352*(K44A) (strain GNE0175) bacteria cultured on agar containing compound 103 at fourfold its MIC. The SpsB mutations were identified by sequencing PCR products.

Together, these data indicate that overexpression of the putative ABC transporter, induced by derepression of its transcription as the result of mutations in the transcriptional repressor *cro*/*cI*, is the main mechanism of *S. aureus* resistance to compound 103.

We then tested the effects of inactivation of the ABC transporter in the absence of *cro*/*cI* mutations. In a neutropenic thigh mouse model of infection, *S. aureus* USA300 with a K44A point mutation in SAUSA300_0352 in a WT *cro*/*cI* background (strain GNE0174) was more sensitive than its parental USA300 WT strain (strain GNE0023) to treatment with a compound 103 analog (see Fig. S3A in the supplemental material). *In vitro*, the K44A mutation had no effect on sensitivity to the compound 103 analog (the MIC was 0.25 µg/ml for both strains). A general phenotypic characterization of the K44A mutant showed no difference from the WT strain with regard to growth rate in Mueller-Hinton broth (MHB) in the absence or presence of serum (see Fig. S3B), sensitivity to antimicrobial peptide protamine (the MIC was 8 µg/ml for both strains) or nisin (the MIC was 200 µg/ml for both strains), or the ability to form biofilm *in vitro* (see Fig. S3C). A recent study showed that transcription of the putative ABC transporter can be transiently induced in WT *S. aureus* (16). Based on those and our observations, we speculate that the putative ABC transporter may become overexpressed in WT *S. aureus* during infection, leading to reduced sensitivity to SpsB inhibition.

### Overexpression of the ABC transporter in the absence of SpsB partially restores viability and protein secretion but not infectivity.

**FIG 3:**
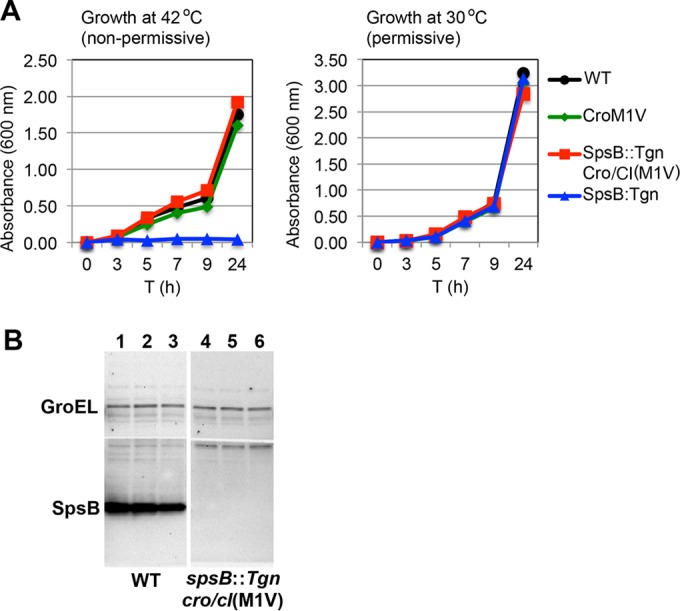
Viability of *S. aureus* USA300 lacking SpsB is restored by *cro*/*cI* mutation *in vitro*. (A) *cro*/*cI* mutation restores *in vitro* growth of a mutant lacking SpsB. At nonpermissive temperature (42°C), plasmid pNL9164-*ltrA* is cured from strain USA300 Δ*mcr spsB*::*Tgn*(pNL9164-*ltrA*) (strain GNE0190) and the TargeTron insertion in *spsB* prevents *spsB* expression, resulting in complete cessation of growth and confirming the essentiality of *spsB*. Remarkably, the growth of this strain is restored by a mutation in *cro*/*cI* [USA300 Δ*mcr spsB*::*Tgn cro*/*cI*(M1V) (strain GNE0191)]. At permissive temperature (30°C), plasmid pNL9164-*ltrA* mediates splicing of the TargeTron insertion that removes it from the *spsB* transcript, allowing SpsB expression and growth of USA300 Δ*mcr spsB*::*Tgn* pNL9164-*ltrA* (strain GNE0190). Circles, USA300 Δ*mcr* (WT; strain GNE0023); triangles, USA300 Δ*mcr spsB*::*Tgn* pNL9164-*ltrA* (strain GNE0190); squares, USA300 Δ*mcr spsB*::*Tgn cro*/*cI*(M1V) (after curing plasmid pNL9164-*ltrA*) (strain GNE0191); diamonds, USA300 Δ*mcr cro*/*cI*(M1V) (strain GNE0117). (B) Western blotting using a polyclonal rabbit anti-SpsB antibody showed a complete lack of SpsB protein in 3 independent whole-cell lysates from USA300 Δ*mcr spsB*::*Tgn cro*/*cI*(M1V) (strain GNE0191) (lanes 4 to 6), compared to normal SpsB expression in USA300 Δ*mcr* (WT, strain GNE0023) (lanes 1 to 3). Rabbit IgG anti-GroEL served as the loading control.

The above-described data showed that overexpression of the putative ABC transporter mediates resistance to the SpsB inhibitor. Therefore, we hypothesized that overexpression of the putative ABC transporter would overcome the essentiality of SpsB for *S. aureus* viability. To test this, we generated a mutant containing an insertion in chromosomal *spsB* and coexpressing a plasmid-encoded LtrA that removes the insertion from transcripts by RNA splicing; this mutant is normally viable at permissive temperature (Fig. 3A, strain GNE0190). Loss of the LtrA-encoding plasmid by culture at a nonpermissive temperature resulted in nonproductive *spsB* transcripts and complete cessation of growth (Fig. 3A), confirming the requirement of SpsB. In the LtrA-containing strain with the *spsB* insertion (strain GNE0190), we then generated a *cro*/*cI*(M1V) mutation by selection with compound 103. After loss of the LtrA-encoding plasmid at the nonpermissive temperature, which was confirmed by culture on antibiotic-selective medium and by plasmid-specific PCR, this USA300 *spsB cro*/*cI* double mutant (GNE0191) showed normal growth compared to that of the WT strain (Fig. 3A). We confirmed both the absence of SpsB protein in this strain (by Western blotting) (Fig. 3B) and overexpression of the ABC transporter (by qRT-PCR) (*SAUSA300_0352* transcript expression was 162- ± 13-fold higher than in the WT USA300 strain). These data demonstrate that *S. aureus* is able to survive without SpsB *in vitro* when the ABC transporter is overexpressed. This is consistent with data from another study describing a strain of *S. aureus* N315 that carries a deletion of *spsB* and a point mutation in a gene homologous to *cro*/*cI* (19).

We next determined the secretion profile of the USA300 *spsB cro*/*cI* double mutant by mass spectrometry. This identified 25 proteins that were released into the culture supernatant without signal peptide cleavage. For 12 of these secreted proteins, an alternative, SpsB-independent cleavage site was identified (see Table S4 in the supplemental material), for which we generated a consensus cleavage motif (see Fig. S4A). Thus, the ABC transporter compensates at least in part for the absence of SpsB by promoting the secretion of a subset of proteins, presumably through an alternative cleavage mechanism.

**FIG 4:**
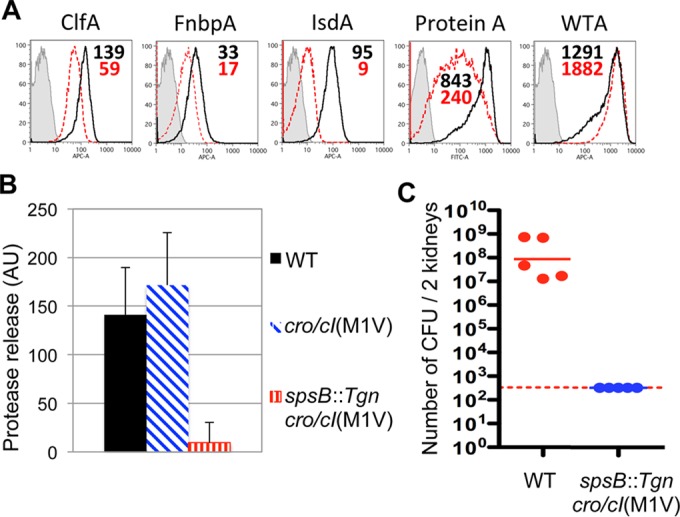
*spsB* *cro*/*cI* double mutant exhibits reduced expression of virulence factors and impaired infectivity. (A) Reduced surface expression of cell wall proteins on *spsB* *cro*/*cI* double mutant. Whole *S. aureus* USA300 WT (black lines, with mean fluorescence intensity [MFI] in black numbers; strain GNE0023) or USA300 Δ*mcr spsB*::*Tgn cro*/*cI*(M1V) double mutant (red dotted lines, with MFI in red numbers; strain GNE0191) bacteria were incubated with MAbs against surface antigens, or WT was incubated with medium alone (gray shading and lines), followed by flow cytometry analysis. The data show significant reductions in surface expression of clumping factor A (ClfA), fibronectin-binding protein A (FnbpA), iron-regulated surface determinant A (IsdA), and protein A, but not of the nonproteinaceous antigen wall teichoic acid (WTA). APC-A, allophycocyanin channel; FITC-A, fluorescein isothiocyanate channel. (B) Defective release of active proteases in culture supernatant of USA300 *spsB*::*Tgn cro*/*cI* double mutant. Filled black, WT USA300 Δ*mcr* (strain GNE0023); hatched blue, USA300 Δ*mcr cro*/*cI*(M1V) (strain GNE0117); vertically striped red, USA300 *spsB*::*Tgn cro*/*cI*(M1V) (strain GNE0191). (C) *spsB*::*Tgn cro*/*cI* double mutant is unable to establish infection. Mice were injected intravenously with *S. aureus* USA300 Δ*mcr* (WT; strain GNE0023) or USA300 Δ*mcr spsB*::*Tgn cro*/*cI*(M1V) (strain GNE0191) double mutant, and numbers of CFU per kidney were determined at day 3 after infection. Dashed line, detection limit.

We then performed a quantitative assessment of the secretion of individual functional proteins in the absence of SpsB. First, the levels of surface expression of several sortase A-anchored proteins, i.e., FnbpA, ClfA, IsdA, and protein A, in the USA300 *spsB cro*/*cI* double mutant were significantly reduced, as shown by flow cytometry (Fig. 4A). Second, the USA300 *spsB cro*/*cI* double mutant demonstrated a severe defect in the release of active proteases (Fig. 4B). All four sortase A-anchored proteins tested contribute to the virulence of *S. aureus* through adherence, iron uptake, and evasion of immune responses, while the secreted proteases facilitate tissue penetration and impair immune responses (20–22). Since the expression of both groups of proteins was quantitatively diminished, we assessed the virulence of this strain in a mouse model of systemic infection. The USA300 *spsB cro/cI* double mutant was completely unable to establish infection (Fig. 4C). Thus, in the absence of SpsB, the overexpression of the ABC transporter restores the secretion of a subset of proteins, likely through an alternative cleavage mechanism. While this allows growth *in vitro*, it does not overcome the requirement of SpsB during infection.

### Concluding remarks.

The present study demonstrates that resistance of the clinical *S. aureus* strain USA300 to compound 103, a potent inhibitor of the type I signal peptidase SpsB, is caused by robust overexpression of a putative ABC transporter, occurring as a result of mutations in *cro*/*cI*. We showed that the *cro*/*cI* gene product functions as a novel transcriptional regulator in *S. aureus*, by repressing the three genes of the putative ABC transporter that are carried in the same operon as *cro*/*cI*. Overexpression of this ABC transporter is both necessary and sufficient to induce resistance to compound 103. A K44A substitution in the predicted ATPase gene of the transporter prevents the development of resistance, indicating that the resistance mechanism involves active transport.

The precise molecular mechanism by which the ABC transporter mediates resistance to SpsB inhibitor compound 103 remains to be determined. The striking observation that, in bacteria lacking SpsB, overexpression of the ABC transporter restores *in vitro* viability and promotes alternative cleavage of secreted proteins suggests that an extracellular protease is linked to transporter activity and resistance. Since the alternative cleavage sites were located N terminal to the SpsB cleavage site, this putative protease may be embedded in the membrane.

Although overexpression of the ABC transporter restored *in vitro* viability of SpsB-deficient *S. aureus* bacteria, it did not rescue the secretion of virulence-associated proteins and *in vivo* virulence in a mouse infection model. This *in vivo* requirement of SpsB may explain its strong conservation as the sole signal peptidase through the evolution of staphylococci as human pathogens.

## MATERIALS AND METHODS

### Bacterial strains and media.

Wild-type clinical *S. aureus* strains were obtained from the Network on Antibiotic Resistance in *Staphylococcus aureus* (NARSA/BEI) program. MRSA strain USA300 refers throughout this paper to strain NRS384 from NARSA/BEI. A global panel of 102 clinical *S. aureus* isolates was selected from the collection of International Health Management Associates, Inc. (IHMA; Schaumburg, IL), based on diversity of geographic locations, antibiotic resistance profiles, and sites of infection. Bacteria were cultured on cation-adjusted Mueller-Hinton agar (MHA) plates or in cation-adjusted Mueller-Hinton broth (MHB). Bacterial strains used in this study are listed in Table S5 in the supplemental material.

### MICs and frequency of resistance.

MICs were determined according to the standard protocol from the Clinical and Laboratory Standards Institute (23). Briefly, *S. aureus* bacteria were harvested from regular MHA plates that had been cultured for ~20 h at 37°C. Serial dilutions of compound 103 were incubated with bacteria at approximately 10^6^ CFU (CFU)/ml in MHB in round-bottom 96 well plates (Corning, Corning, NY) for 22 h at 37°C. MICs were determined by the lowest concentration of compound 103 showing absence of growth by visual inspection.

To determine the frequency of spontaneous resistance, suspensions containing approximately 5 × 10^9^ CFU of *S. aureus* were cultured on MHA plates containing compound 103 at fourfold the MIC; serial dilutions were cultured in parallel on regular MHA to determine the actual number of CFU (input). The frequency of spontaneous resistance was defined as the number of viable colonies able to grow in the presence of compound 103 after culture at 37°C for 2 days (output) divided by input.

### Mutations and genetic engineering of *S. aureus*.

Compound 103-resistant mutants derived from *S. aureus* USA300 were selected after culture on MHA containing 4 µg/ml of compound 103 for 2 days. Single colonies were restreaked onto MHA with 4 µg/ml of compound 103 to confirm resistance and establish clonality.

For transformations, we used the methylation-deficient Δ*mcr* strain of USA300 (strain GNE0023), which exhibits improved transformability while retaining unaltered *in vivo* virulence (24). To generate mutations in *SAUSA300_0351*, _*0352*, and _*0353* genes by homologous recombination, we transformed USA300 Δ*mcr* using targeting constructs created in the vector pIMAY (24). To generate mutations in *SAUSA300_0351*, _*0352*, and _*0353* genes in a *cro*/*cI* mutant background, we selected colonies from USA300 Δ*mcr* cultured on plates containing fourfold the MIC of compound 103. Sequencing of PCR products of the *cro*/*cI* gene from resistant colonies yielded a clone with a Q16stop codon mutation in *cro*/*cI* (strain GNE0173). We further mutated *SAUSA300_0351*, _*0352*, and _*0353* in this USA300 Δ*mcr cro*/*cI* (Q16stop) clone to study the contributions of these three genes to *cro*/*cI*-mediated resistance.

A conditional SpsB-deficient strain was generated by inserting a TargeTron (Tgn; a *Lactococcus lactis* LI.LtrB group II intron) into the *spsB* gene and coexpressing a temperature-sensitive plasmid, pNL9164-*ltrA*, that encodes LtrA (Sigma, St. Louis, MO) (25) in USA300 Δ*mcr* (clone GNE0190). In the presence of LtrA, the LtrB group II intron is spliced out of *spsB* mRNA, enabling the synthesis of functional SpsB protein. In this clone, we generated a mutation in *cro*/*cI* (i.e., M1V) by selection on agar containing compound 103 at a concentration of fourfold the MIC. Plasmid pNL9164-*ltrA* was cured out by culture at nonpermissive temperature (42°C), which was confirmed by absence of growth on antibiotic-selective medium and by plasmid-specific PCR, and which generated USA300 Δ*mcr spsB*::*Tgn cro*/*cI*(M1V) (clone GNE0191).

A strain of USA300 Δ*mcr* overexpressing SpsB (USA300 SpsB-high; GNE0028) was engineered by cloning SpsB under the constitutive *sarA* promoter into vector pMK4, resulting in SpsB expression that was ~20-fold higher than the wild-type level as determined by Western blotting. A USA300 Δ*mcr* strain that underexpresses SpsB (USA300 SpsB-low; GNE0028) was generated by genomic incorporation of *spsB* under the isopropyl-β-d-thiogalactopyranoside (IPTG)-sensitive promoter *spac*; without IPTG, this strain expresses SpsB at ~5% of the wild-type level as determined by Western blotting.

### RNA isolation and transcript analysis by qRT-PCR.

To isolate *S. aureus* RNA, ~10^8^ CFU from a 22-h culture in MHB were treated with RNAprotect bacterial reagent (Qiagen, Valencia, CA) for 10 min at room temperature and lysed for 30 min at 37°C in 30 mM Tris (pH 8.0) containing 1 mM EDTA, 1 mg/ml of lysostaphin (Sigma), and protease K (Qiagen), followed by mechanical disruption using a bead beater (BioSpec Products, Bartlesville, OK). RNA was purified using an RNeasy RNA purification kit (Qiagen); RNA concentrations were determined using a NanoDrop 8000 instrument (Thermo Scientific, Waltham, MA).

Transcript levels were analyzed by qRT-PCR as described previously (26). Briefly, purified RNA was treated with DNase (DNA-free kit; Ambion) and reverse transcribed using gene-specific reverse primers and the Superscript III reverse transcription kit (Invitrogen, Carlsbad, CA), followed by PCRs with gene-specific primers, 6-carboxyfluorescein (FAM)-labeled probes (see Table S6 in the supplemental material), and TaqMan universal PCR master mix (Applied Biosystems). PCRs were recorded using the 7500 real-time PCR system (Applied Biosystems). Delta cycle threshold (Δ*C_T_*) values were determined by subtracting the *C_T_* values of the rRNA gene of *S. aureus* (*rrsA*), used for normalization, from the *C_T_* value of each test gene. Data were expressed as the fold change (FC) of transcript expression in mutants relative to the expression of the transcript in wild-type bacteria and were calculated using the following formula: FC = 2^[Δ*C_T_* (wild-type) − Δ*C_T_* (mutant)].

### Quantitative analysis of cell wall protein expression and secretion of active proteases.

Human IgG1 monoclonal antibodies (MAbs) against *S. aureus* fibronectin-binding protein A (FnbpA; clone 6931), clumping factor A (ClfA; clone 4675), iron-regulated surface determinant A (IsdA; clone 4402), and wall teichoic acid (WTA; clone 7574) were cloned from peripheral B cells of patients after *S. aureus* infection using the Symplex technology (27–29). Murine IgG2a against protein A (MAb 8F9) was generated by immunizing BALB/c mice with USA300 bacteria. To quantify surface protein expression, approximately 10^7^ bacteria were incubated with 200 µg/ml of rabbit IgG (Sigma, St. Louis, MO), 2 µg/ml of MAbs against surface antigens, and fluorochrome-labeled anti-human or anti-mouse IgG *F*(ab′)_2_ (Jackson ImmunoResearch, West Grove, PA). Fluorescence was analyzed using a fluorescence-activated cell sorter (FACSAria; Becton, Dickinson) and FlowJo software (version 8.4.5), and the fluorescence intensity served as a measure for surface expression of cell wall proteins.

To quantify the secretion of active proteases, culture supernatants from bacteria cultured in MHB at 37°C for 6 h were centrifuged at 2,000 × *g* for 10 min and passaged through a 0.2-µm filter. The amounts of active proteases released into the culture supernatants were quantified using the EnzCheck assay kit (Invitrogen), which detects the activity of multiple classes of proteases based on fluorescent casein cleavage.

### Mouse infection.

For systemic infection, 7-week-old female A/J mice (Jackson, Bar Harbor, ME) were injected intravenously through the tail vein with 100 µl phosphate-buffered saline (PBS) containing approximately 2 × 10^6^ CFU of log-phase *S. aureus*. At 2, 4, or 7 days after infection, kidneys were harvested and homogenized using a gentleMACS dissociator (Miltenyi Biotec, Inc., San Diego, CA), and serial dilutions were plated on agar to determine the number of CFU per organ. Mouse experiments were approved by the Institutional Animal Care and Use Committee and conducted in an AAALACi-accredited facility.

## SUPPLEMENTAL MATERIAL

Text S1  Supplemental materials and methods. Download Text S1, DOC file, 0.1 MB

Figure S1  Compound 103: structure and inhibition of SpsB enzyme activity. Chemical structure of (A) compound 103 and (B) arylomycin A16. (C) Compound 103 inhibits enzymatic activity of purified SpsB with a calculated 50% inhibitory concentration (IC_50_) of 6.86 ± 0.27 nM. All values are the average ± SD from three independent experiments. Download Figure S1, TIF file, 0.3 MB

Figure S2  USA300 *cro*/*cI* mutant bacteria are normally virulent in mice. A/J mice were infected with *S. aureus* USA300 Δ*mcr* (WT; strain GNE0023) (left two panels) or USA300 Δ*mcr cro*/*cI*(T29N) (strain GNE0219) (right two panels). At 2 (D2) and 7 (D7) days after infection, the numbers of CFU in the kidneys were determined. The *cro*/*cI* mutation was not associated with a significant *in vivo* fitness cost as determined by the Mann-Whitney *t* test (*P* > 0.05). Dotted line, detection limit. Download Figure S2, TIF file, 0.3 MB

Figure S3  Inactivation of the putative ABC transporter sensitizes *S. aureus* to SpsB inhibition *in vivo*. (A) Neutropenic mice were given an intramuscular injection with 2 × 10^5^ CFU of *S. aureus* and treated by continuous infusion with PBS (0) or with two different doses of compound 103 analog, which inhibits SpsB, reaching a plasma concentration at steady state (*C*_ss_) of either 2 or 8 µg/ml. One day after infection, the numbers of viable bacteria in the thighs were determined. The *SAUSA300_0352*(K44A) point mutant with inactive ABC transporter in a WT *cro*/*cI* background showed a lower bacterial burden in the thigh muscle than did its parental WT strain. The MICs of the compound 103 analog were equal for both strains (0.25 µg/ml). The dotted line represents the number of bacteria in the inoculum. (B) The *SAUSA300_0352*(K44A) mutant shows no difference from the WT strain (GNE0023) in growth rate when cultured at 37°C in MHB either in the absence (left) or presence (right) of 25% normal human serum (NHS). (C) The ability to form biofilm *in vitro* was similar for both strains. WT, *S. aureus* USA300 WT (strain GNE0023); K44A, S*. aureus* USA300 *SAUSA300_0352*(K44A) mutant (strain GNE0174). Download Figure S3, TIF file, 0.5 MB

Figure S4  SpsB-independent protein secretion. Supernatants of *S. aureus* USA300 *spsB*::*Tgn cro*/*cI*(M1V) double mutant (strain GNE0191) and of WT USA300 Δ*mcr* (strain GNE0023) were harvested from 18-h cultures grown at 37°C in MHB and analyzed by mass spectrometry and gel electrophoresis. (A) Logogram showing an alternative cleavage site identified by mass spectrometry in proteins secreted independently of SpsB in the *spsB cro*/*cI* double mutant (strain GNE0191). The alternative cleavage site appeared at various positions between 13 and 22 (N terminal to the signal peptide) in 12 of 25 secreted proteins (listed in Table S4 in the supplemental material). (B) Gel image of secreted proteins. Supernatants were separated by ultrafiltration using 10,000 nominal molecular weight limit (NMWL) cellulose filters into flowthrough and 20-fold-concentrated retentate fractions. Retentates (estimated to be 25 µg/lane) were fractionated by SDS-PAGE, and the gel was stained with Coomassie blue G-250. Lane 1, MW marker; lane 2, WT USA300 Δ*mcr* (strain GNE0023); lane 3, USA300 Δ*mcr spsB*::*Tgn cro*/*cI*(M1V) (strain GNE0191). This image shows that the secretion profile of the *spsB cro*/*cI* double mutant strain is generally different from that of the WT. Download Figure S4, TIF file, 0.9 MB

Table S1  *cro*/*cI* nucleotide and amino acid changes leading to reduced sensitivity to compound 103. Shown are all mutations found inside or upstream of the *SAUSA300_0350* (*cro*/*cI*) gene. Each of these mutations resulted in an increase in the MIC for compound 103 by at least 16-fold compared to the MIC for USA300 WT. Table S1, DOCX file, 0.1 MB

Table S2  *cro*/*cI*(M1V) mutant of *S. aureus* USA300 exhibits unaltered sensitivity to antibiotics of various classes. Table S2, DOCX file, 0.1 MB

Table S3  Genes that are overexpressed by at least twofold in the *cro*/*cI*(M1V) mutant of USA300 (strain GNE0117) compared to their expression in WT USA300 by RNA sequencing. The essential role of the clearly outstanding top four genes, which are organized in an operon with *cro*/*cI* itself, in resistance to compound 103 was confirmed experimentally by mutagenesis analysis (this study). For more details, see the text and Fig. 2A Table S3, DOCX file, 0.1 MB

Table S4  SpsB-independent processing of secreted proteins. Proteins secreted into the culture supernatants of *S. aureus* WT USA300 Δ*mcr* (strain GNE0023) and USA300 Δ*mcr spsB*::*Tgn cro*/*cI*(M1V) (strain GNE0191), cultured for 18 h at 37°C in MHB, were analyzed by mass spectrometry. Proteins detected in the supernatants of USA300 Δ*mcr spsB*::*Tgn cro*/*cI*(M1V) were secreted independently of SpsB. SpsB cleavage sites in proteins secreted by WT USA300 Δ*mcr* were confirmed either by the presence of a peptide whose N terminus matched the right flanking sequence in the trypsin digest of secreted proteins retained by a 10-kDa filter or by a peptide with the matching C-terminal sequence identified in the filter’s flowthrough. The observed N- or C-terminal flanking residues are indicated in boldface. For the first 12 proteins in this list, we predicted a consensus cleavage motif (see Fig. S4A in the supplemental material). Table S4, DOCX file, 0.1 MB

Table S5  Bacterial strains and plasmids. Table S5, DOC file, 0.1 MB

Table S6  Primers and probes used for qRT-PCR analysis of gene expression. Shown are the sequences of the forward and reverse primers and probes used to determine the expression of the corresponding genes, indicated in the left column, by qRT-PCR (see Fig. 3B). Table S6, DOCX file, 0.1 MB

## References

[1] Servick K 2015 The drug push. Science 348:850–853. doi:10.1126/science.348.6237.850. 25999488

[2] DeLeo FR, Chambers HF 2009 Reemergence of antibiotic-resistant Staphylococcus aureus in the genomics era. J Clin Invest 119:2464–2474. doi:10.1172/JCI38226. 19729844 PMC2735934

[3] Small PM, Chambers HF 1990 Vancomycin for Staphylococcus aureus endocarditis in intravenous drug users. Antimicrob Agents Chemother 34:1227–1231. doi:10.1128/AAC.34.6.1227. 2393284 PMC171789

[4] Roemer T, Boone C 2013 Systems-level antimicrobial drug and drug synergy discovery. Nat Chem Biol 9:222–231. doi:10.1038/nchembio.1205. 23508188

[5] Smitha Rao CV, Anné J 2011 Bacterial type I signal peptidases as antibiotic targets. Future Microbiol 6:1279–1296. doi:10.2217/fmb.11.109. 22082289

[6] Van Roosmalen ML, Geukens N, Jongbloed JD, Tjalsma H, Dubois JY, Bron S, van Dijl JM, Anné J 2004 Type I signal peptidases of Gram-positive bacteria. Biochim Biophys Acta 1694:279–297. doi:10.1016/j.bbamcr.2004.05.006. 15546672

[7] Dalbey RE, Wickner W 1985 Leader peptidase catalyzes the release of exported proteins from the outer surface of the Escherichia coli plasma membrane. J Biol Chem 260:15925–15931. 2999144

[8] Smith PA, Romesberg FE 2012 Mechanism of action of the arylomycin antibiotics and effects of signal peptidase I inhibition. Antimicrob Agents Chemother 56:5054–5060. doi:10.1128/AAC.00785-12. 22802255 PMC3457390

[9] Therien AG, Huber JL, Wilson KE, Beaulieu P, Caron A, Claveau D, Deschamps K, Donald RG, Galgoci AM, Gallant M, Gu X, Kevin NJ, Lafleur J, Leavitt PS, Lebeau-Jacob C, Lee SS, Lin MM, Michels AA, Ogawa AM, Painter RE, Parish CA, Park YW, Benton-Perdomo L, Petcu M, Phillips JW, Powles MA, Skorey KI, Tam J, Tan CM, Young K, Wong S, Waddell ST, Miesel L 2012 Broadening the spectrum of beta-lactam antibiotics through inhibition of signal peptidase type I. Antimicrob Agents Chemother 56:4662–4670. doi:10.1128/AAC.00726-12. 22710113 PMC3421906

[10] Schimana J, Gebhardt K, Holtzel A, Schmid DG, Sussmuth R, Muller J, Pukall R, Fiedler HP 2002 Arylomycins A and B, new biaryl-bridged lipopeptide antibiotics produced by Streptomyces sp. Tu 6075. I. Taxonomy, fermentation, isolation and biological activities. J Antibiot (Tokyo) 55:565–570. doi:10.7164/antibiotics.55.565. 12195962

[11] Höltzel A, Schmid DG, Nicholson GJ, Stevanovic S, Schimana J, Gebhardt K, Fiedler H, Jung G 2002 Arylomycins A and B, new biaryl-bridged lipopeptide antibiotics produced by streptomyces sp. Tu 6075. II. Structure elucidation. J Antibiot (Tokyo) 55:571–577. doi:10.7164/antibiotics.55.571. 12195963

[12] Luo C, Roussel P, Dreier J, Page MG, Paetzel M 2009 Crystallographic analysis of bacterial signal peptidase in ternary complex with arylomycin A2 and a beta-sultam inhibitor. Biochemistry 48:8976–8984. doi:10.1021/bi9009538. 19655811

[13] Smith PA, Powers ME, Roberts TC, Romesberg FE 2011 In vitro activities of arylomycin natural-product antibiotics against Staphylococcus epidermidis and other coagulase-negative staphylococci. Antimicrob Agents Chemother 55:1130–1134. doi:10.1128/AAC.01459-10. 21189343 PMC3067118

[14] Johnson AD, Poteete AR, Lauer G, Sauer RT, Ackers GK, Ptashne M 1981 Lambda repressor and cro—components of an efficient molecular switch. Nature 294:217–223. doi:10.1038/294217a0. 6457992

[15] Oppenheim AB, Kobiler O, Stavans J, Court DL, Adhya S 2005 Switches in bacteriophage lambda development. Annu Rev Genet 39:409–429. doi:10.1146/annurev.genet.39.073003.113656. 16285866

[16] Craney A, Romesberg FE 2015 A putative Cro-like repressor contributes to arylomycin resistance in Staphylococcus aureus. Antimicrob Agents Chemother 59:3066–3074. doi:10.1128/AAC.04597-14. 25753642 PMC4432125

[17] Matveeva EA, He P, Whiteheart SW 1997 N-ethylmaleimide-sensitive fusion protein contains high and low affinity ATP-binding sites that are functionally distinct. J Biol Chem 272:26413–26418. doi:10.1074/jbc.272.42.26413. 9334216

[18] Hanson PI, Whiteheart SW 2005 AAA+ proteins: have engine, will work. Nat Rev Mol Cell Biol 6:519–529. doi:10.1038/nrm1684. 16072036

[19] Craney A, Dix MM, Adhikary R, Cravatt BF, Romesberg FE 2015 An alternative terminal step of the general secretory pathway in Staphylococcus aureus. mBio 6:e01178-15. doi:10.1128/mBio.01178-15. 26286693 PMC4542188

[20] Cheng AG, Kim HK, Burts ML, Krausz T, Schneewind O, Missiakas DM 2009 Genetic requirements for Staphylococcus aureus abscess formation and persistence in host tissues. FASEB J 23:3393–3404. doi:10.1096/fj.09-135467. 19525403 PMC2747682

[21] Dubin G 2002 Extracellular proteases of Staphylococcus spp. Biol Chem 383:1075–1086. doi:10.1515/BC.2002.116. 12437090

[22] Thammavongsa V, Kim HK, Missiakas D, Schneewind O 2015 Staphylococcal manipulation of host immune responses. Nat Rev Microbiol 13:529–543. doi:10.1038/nrmicro3521. 26272408 PMC4625792

[23] Clinical and Laboratory Standards Institute. 2007 Performance standards for antimicrobial susceptibility testing; 17th informational supplement. CLSI document M100-S17 Clinical and Laboratory Standards Institute, Wayne, PA.

[24] Monk IR, Shah IM, Xu M, Tan MW, Foster TJ 2012 Transforming the untransformable: application of direct transformation to manipulate genetically *Staphylococcus aureus* and *Staphylococcus epidermidis*. mBio 3:e00277-11. doi:10.1128/mBio.00277-11. 22434850 PMC3312211

[25] Yao J, Zhong J, Fang Y, Geisinger E, Novick RP, Lambowitz AM 2006 Use of targetrons to disrupt essential and nonessential genes in Staphylococcus aureus reveals temperature sensitivity of Ll.LtrB group II intron splicing. RNA 12:1271–1281. doi:10.1261/rna.68706. 16741231 PMC1484445

[26] Date SV, Modrusan Z, Lawrence M, Morisaki JH, Toy K, Shah IM, Kim J, Park S, Xu M, Basuino L, Chan L, Zeitschel D, Chambers HF, Tan MW, Brown EJ, Diep BA, Hazenbos WL 2014 Global gene expression of methicillin-resistant Staphylococcus aureus USA300 during human and mouse infection. J Infect Dis 209:1542–1550. doi:10.1093/infdis/jit668. 24286981

[27] Meijer PJ, Andersen PS, Haahr Hansen M, Steinaa L, Jensen A, Lantto J, Oleksiewicz MB, Tengbjerg K, Poulsen TR, Coljee VW, Bregenholt S, Haurum JS, Nielsen LS 2006 Isolation of human antibody repertoires with preservation of the natural heavy and light chain pairing. J Mol Biol 358:764–772. doi:10.1016/j.jmb.2006.02.040. 16563430

[28] Meijer PJ, Nielsen LS, Lantto J, Jensen A 2009 Human antibody repertoires. Methods Mol Biol 525:261–277. doi:10.1007/978-1-59745-554-1_13. 19252857

[29] Hazenbos WL, Kajihara KK, Vandlen R, Morisaki JH, Lehar SM, Kwakkenbos MJ, Beaumont T, Bakker AQ, Phung Q, Swem LR, Ramakrishnan S, Kim J, Xu M, Shah IM, Diep BA, Sai T, Sebrell A, Khalfin Y, Oh A, Koth C, Lin SJ, Lee BC, Strandh M, Koefoed K, Andersen PS, Spits H, Brown EJ, Tan MW, Mariathasan S 2013 Novel staphylococcal glycosyltransferases SdgA and SdgB mediate immunogenicity and protection of virulence-associated cell wall proteins. PLoS Pathog 9:e1003653. doi:10.1371/journal.ppat.1003653. 24130480 PMC3794999

