# Supplementary material for: A Putative Bacterial ABC Transporter Circumvents the Essentiality of Signal Peptidase
Source: mBio. 2016 Sep 6;7(5):e00412-16. doi: 10.1128/mBio.00412-16 (PMC5013292; doi:10.1128/mBio.00412-16)
Supplement: Text S1 — Supplemental materials and methods. Download [file mbo004162962s1.doc]

**SUPPLEMENTARY MATERIALS AND METHODS**

**Synthesis of SpsB inhibitor compound 103.**

Compound 103 is a close analog of the previously described compound M131 . It was prepared according to procedures described in US 20140142029 as follows: ^1^H NMR (400 MHz, CD_3_OD) δ 8.67, (d, J = 8.8 Hz, 1H), 7.87 (d, J = 8.4 Hz, 2H), 7.70 (d, J = 8.4 Hz, 2H), 7.56 (d, J = 8.0 Hz, 2H), 7.28 (d, J = 8.0 Hz, 2H), 7.06 – 7.09 (m, 2H), 6.95 – 7.00 (m, 2H), 6.94 (d, J = 8.4 Hz, 1H), 6.86 (d, J = 8.4 Hz, 1H), 6.43 (s, 1H), 4.75 – 4.90 (m, 1H), 3.65 (dd, J = 13.4, 7.8 Hz, 1H), 3.35 – 3.45 (m, 2H), 3.05 – 3.15 (m, 1H), 2.87 (s, 3H), 2.75 – 2.85 (m, 3H), 2.65 (t, J = 7.8 Hz, 2H), 1.75 – 1.85 (m, 1H), 1.55 – 1.70 (m, 6H), 1.40 1.50 (m, 5H), 1.36 (d, J = 6.8 Hz, 3H), 1.15 (d, J = 7.2, 3H), 0.95 (t, J = 7.2 Hz, 3H). MS (ESI) for (C_48_H_58_N_6_O_9_): *m/z* 864.3 (M + H)^+^.

**Enzymatic activity of recombinant SpsB.**

White 384-well low-volume polypropylene assay plates (Thermo Scientific, Waltham, MA) were pre-spotted with 25 nL per well of compound 103, serially diluted in DMSO, using an Echo instrument (Labcyte, Sunnyvale, CA). A 10 μL reaction in PBS (pH 7.4) containing 0.1% Triton X-100 was then initiated by adding 500 pM full length recombinant SpsB from *S. aureus* plus 10 μM fluorogenic peptide substrate Dabcyl-βAla-KPAKAAE-Edans (GL Biochem, Boston, MA). Substrate cleavage separates the Dabcyl quencher from the Edans fluorophore, resulting in 490nm fluorescence (340nm excitation). The reaction was read kinetically using an M-1000 plate reader (Tecan, Morrisville, NC), plotting the initial slope (rate) of fluorescence generation versus inhibitor concentration to derive IC_50_ values.

**Whole-genome DNA sequencing.**

For genomic DNA isolation, *S. aureus* bacteria were harvested from overnight cultures in MHB and lysed in 60 μg/mL of lysostaphin (Sigma) for 15 min at 37 ^o^C; DNA was purified using the Wizard genomic DNA purification kit (Promega, Madison, WI).

Samples of 1 μg of genomic *S. aureus* DNA were sheared to 200 ~ 300 base pair fragments using an E220 acoustic shearing device (Covaris, Woburn, MA). Libraries were made using TruSeq Nano DNA Library Preparation Kit (Illumina). Fragments were end-repaired and an adenosine residue was ligated to the 3’ end of the fragment. Dual index multiplexing adapter oligonucleotides were added to the fragments for sequencing. Sample libraries were quantified using Kapa library quantification kit (Kapa Biosciences, Woburn, MA), pooled, adjusted to 2 nM, and denatured in 0.05 M NaOH, followed by dilution to 2 pM in pre-chilled HT1 buffer. Paired-end sequencing (100 bp) was performed using Rapid flow cell on HiSeq2500 (Illumina), to yield 2M reads per sample.

**Transcriptome analysis by RNA sequencing.**

For whole-genome RNA sequencing, bacterial RNA was purified as described in the methods section "RNA isolation and transcript analysis by qRT-PCR" in the main body of the manuscript. The integrity of RNA samples was determined using the 2100 Bioanalyzer (Agilent Technologies, Santa Clara, CA). Samples of 0.1 - 1 μg of total bacterial RNA were depleted of ribosomal RNA using the Ribo-Zero rRNA Removal Kit (Bacteria) (Illumina, San Diego, CA). Recovered samples were further purified using Agencourt RNA Clean XP Beads (Beckman Coulter, Inc., Indianapolis, IN), and TruSeq RNA Sample Preparation Kit v2 (Illumina) was used starting at the fragmentation step. Size of the libraries was confirmed using Fragment Analyzer (Advanced Analytical Technologies, Ames, IA) and their concentration was determined by qPCR based method using Kapa quantification kit (Kapa Biosystems, Wilmington, MA). Libraries were multiplexed and then sequenced on Illumina HiSeq2500 (Illumina) to generate 30M of single end 50 base pair reads. All sequencing reads were evaluated for quality using the Bioconductor ShortRead package . Genes were identified by aligning RNA-Seq reads to the bacterial genome of *S. aureus* USA300 NRS384 using a customized analysis pipeline based on the GSNAP/GMAP package . Expression counts per gene were obtained by counting the number of reads aligned uniquely to each gene locus as defined by NCBI and Ensembl gene annotations and RefSeq mRNA sequences. Differential gene expression analysis was performed using DESeq2 algorithm .

**Analysis of SpsB expression by western blotting.**

*S. aureus* bacteria were lysed in 120 mM NaCl and 50 mM Tris-HCl (pH 8.0) containing 0.1% NP-40 and protease inhibitor cocktail (Roche, Basel, Switzerland) for 30 min on ice, followed by mechanical disruption using a bead beater (Biospec Products, Bartlesville, OK). Proteins were normalized using BCA protein assay kit (Thermo Scientific), loaded at 1 μg per lane, separated on a 4-12% Tris-glycine gel (BioRad, Hercules, CA), and transferred to nitrocellulose membrane (Invitrogen). Membranes were blotted with 1 μg/mL of polyclonal rabbit antibodies against SpsB (affinity-purified from serum of rabbits immunized with *Escherichia coli* expressed recombinant USA300 SpsB) or loading control GroEL (Stressgen Biotechnologies, San Diego, CA) in TBS containing 2% BSA and 5 μg/mL of human IgG-Fc (Bethyl Laboratories, Montgomery, TX), followed by detection using peroxidase-conjugated anti-rabbit secondary antibodies (Jackson Immunoresearch, West Grove, PA).

**Mass spectrometry analysis of protein secretion profiles.**

Supernatants of *S. aureus* WT USA300 Δ*mcr* (GNE0023) and USA300 Δ*mcr*; *spsB:Tgn*; *cro/cI* (M1V) (GNE0191), cultured for 18 h at 37 ^o^C in MHB (late exponential phase), were separated by ultrafiltration using Amicon 10,000 NMWL cellulose filters (EMD Millipore; Billerica, MA) into flow-through and 20-fold concentrated retentate fractions. Retentates were fractionated by SDS-PAGE. The gel was stained with Coomassie Blue G-250 (Supplementary Figure S4B) and each gel lane was excised top-to-bottom into 6 bands. Reduction, alkylation, and trypsin digestion of the proteins was performed as previously described . Peptides collected in the ultrafiltration flow-through were acidified with trifluoroacetic acid, concentrated and desalted by C18 Sep-Pak cartridge (500mg sorbent; Waters Inc.), lyophilized, and reconstituted in water for mass spectrometric analysis. Digested samples were analyzed by capillary reverse phase chromatography- electrospray tandem mass spectrometry on an Orbitrap mass spectrometer in data-dependent acquisition mode (“top 15” high/low resolution for MS and MS/MS respectively) as described . Tandem mass spectral results were submitted for database searching using the Mascot search algorithm (Matrix Sciences) against a concatenated target-decoy database of human proteins and common contaminants. Semi-tryptic specificity with two allowed missed cleavages and variable modifications of cysteine carbamidomethylation and methionine oxidation were stipulated for the data derived from digested gel bands, or without enzyme specify and only Met-oxidation as a variable modification for the flow-through sample data. Peptide spectral matches were filtered to a false discovery rate (FDR) of 1% using a linear discriminant algorithm (LDA) . Label-free relative quantitation of proteins across the samples was accomplished by spectral counting (ref) . Sample loads were normalized to equal amounts by weight, and since the total number of peptides identified in each sample varied by less than 10%, no further normalization was performed. The average number of hits for cytoplasmic ribosomal proteins (a total of 53 proteins containing "ribosomal" in their annotation) found in the supernatant did not significantly differ between the two *S. aureus* strains analyzed (average no. of hits being 5.7 for *S. aureus* WT USA300 Δ*mcr* GNE0023, and 6.7 for USA300 Δ*mcr*; *spsB:Tgn*; *cro/cI* (M1V) GNE0191), minimizing the possible effect of lysis of bacteria on the secretion profiles.

***In vitro* biofilm formation.**

*S. aureus* from a single colony was cultured for 3 h to log phase, followed by inoculation of 5x10^5^ CFU/mL in 150 μL tryptic soy broth + 5% glucose (TSBG) on Calgary devices (Innovotech, Edmonton, Canada) as described . The devices were incubated overnight at 37°C, followed by 3 washes in 180 μL PBS, immersion in 180 μL of recovery buffer (PBS + 20 mg/mL of saponin + 10 mg/mL TritonX-80), and sonication using 3510 ultrasonic bath (Branson, Danbury, CT) for 30 minutes. Viable biofilm-associated bacteria were enumerated by serial dilution and CFU plating.

**Neutropenic thigh model of *S. aureus* infection in mice.**

Five and two days prior to infection, jugular vein cannulated CD-1 mice (Charles River Laboratories, Hollister, CA) were induced neutropenic by intraperitoneal injection of 150 and 100 mg/kg cyclophosphamide, respectively. One day before infection, saline was infused intravenously at a rate of 20 μL/h for 24 hours using PHD Ultra infuse pump (Harvard Apparatus, Holliston, MA). Mice were infected by intramuscular injection of 2x10^5^ CFU of *S. aureus* into the thigh muscle. Starting one h after infection, mice were infused intravenously with different concentrations of SpsB inhibitor or PBS as control at a rate of 80 uL/h for 23 h. Twenty-four h after infection, thigh muscles were homogenized using a gentleMACS Dissociator (Miltenyi Biotec Inc.; San Diego, CA). Serial dilutions were plated on agar to determine the number of CFU per organ. Mouse experiments were approved by the Institutional Animal Care and Use Committee and conducted in an AAALACi-accredited facility.

**REFERENCES**

1. **Therien AG, Huber JL, Wilson KE, Beaulieu P, Caron A, Claveau D, Deschamps K, Donald RG, Galgoci AM, Gallant M, Gu X, Kevin NJ, Lafleur J, Leavitt PS, Lebeau-Jacob C, Lee SS, Lin MM, Michels AA, Ogawa AM, Painter RE, Parish CA, Park YW, Benton-Perdomo L, Petcu M, Phillips JW, Powles MA, Skorey KI, Tam J, Tan CM, Young K, Wong S, Waddell ST, Miesel L.** 2012. Broadening the spectrum of beta-lactam antibiotics through inhibition of signal peptidase type I. Antimicrobial agents and chemotherapy **56:**4662-4670.

2. **Morgan M, Anders S, Lawrence M, Aboyoun P, Pages H, Gentleman R.** 2009. ShortRead: a bioconductor package for input, quality assessment and exploration of high-throughput sequence data. Bioinformatics **25:**2607-2608.

3. **Wu TD, Nacu S.** 2010. Fast and SNP-tolerant detection of complex variants and splicing in short reads. Bioinformatics **26:**873-881.

4. **Anders S, Huber W.** 2010. Differential expression analysis for sequence count data. Genome biology **11:**R106.

5. **Diep BA, Phung Q, Date S, Arnott D, Bakalarski C, Xu M, Nakamura G, Swem DL, Alexander MK, Le HN, Mai TT, Tan MW, Brown EJ, Nishiyama M.** 2014. Identifying potential therapeutic targets of methicillin-resistant Staphylococcus aureus through in vivo proteomic analysis. The Journal of infectious diseases **209:**1533-1541.

6. **Elias JE, Gygi SP.** 2010. Target-decoy search strategy for mass spectrometry-based proteomics. Methods in molecular biology **604:**55-71.

7. **Liu H, Sadygov RG, Yates JR, 3rd.** 2004. A model for random sampling and estimation of relative protein abundance in shotgun proteomics. Anal Chem **76:**4193-4201.

8. **Ceri H, Olson ME, Stremick C, Read RR, Morck D, Buret A.** 1999. The Calgary Biofilm Device: new technology for rapid determination of antibiotic susceptibilities of bacterial biofilms. J Clin Microbiol **37:**1771-1776.
