# Supplementary figures and images for: A Putative Bacterial ABC Transporter Circumvents the Essentiality of Signal Peptidase
Source: mBio. 2016 Sep 6;7(5):e00412-16. doi: 10.1128/mBio.00412-16 (PMC5013292; doi:10.1128/mBio.00412-16)

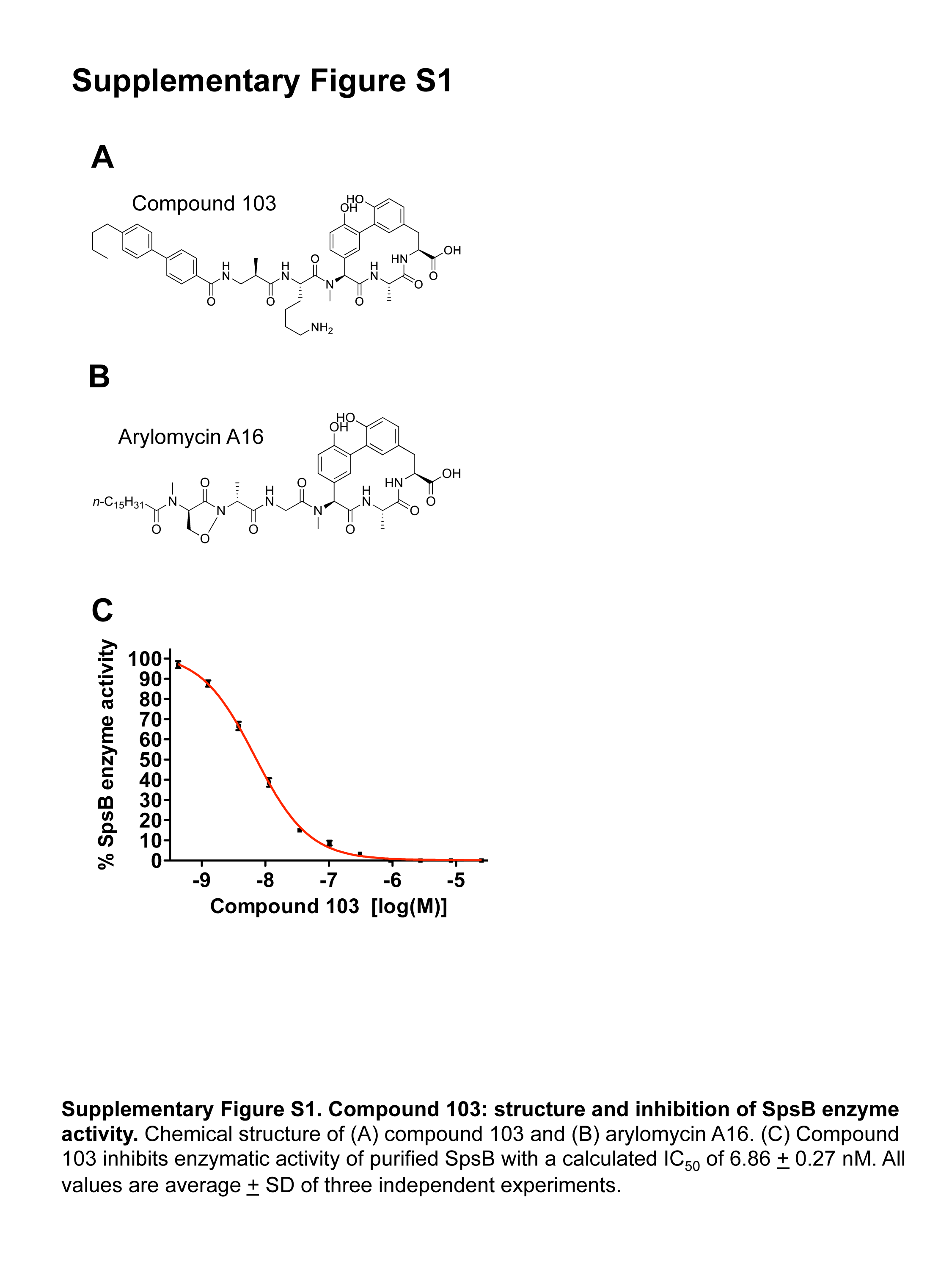

Supplement: Figure S1 — Compound 103: structure and inhibition of SpsB enzyme activity. Chemical structure of (A) compound 103 and (B) arylomycin A16. (C) Compound 103 inhibits enzymatic activity of purified SpsB with a calculated 50% inhibitory concentration (IC50) of 6.86 ± 0.27 nM. All values are the average ± SD from three independent experiments. Download [file mbo004162962sf1.tif]

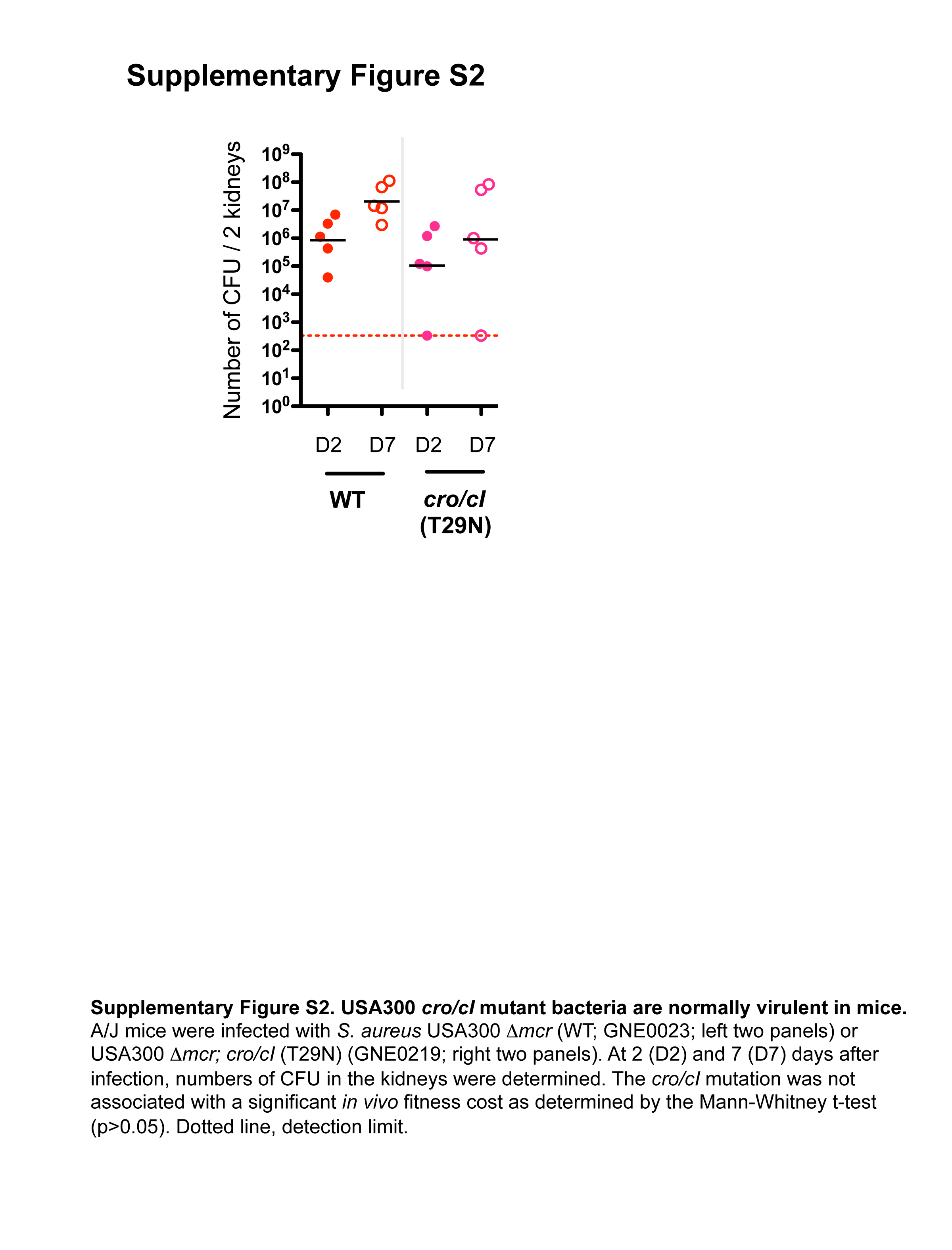

Supplement: Figure S2 — USA300 cro/cI mutant bacteria are normally virulent in mice. A/J mice were infected with S. aureus USA300 Δmcr (WT; strain GNE0023) (left two panels) or USA300 Δmcr cro/cI(T29N) (strain GNE0219) (right two panels). At 2 (D2) and 7 (D7) days after infection, the numbers of CFU in the kidneys were determined. The cro/cI mutation was not associated with a significant in vivo fitness cost as determined by the Mann-Whitney t test (P > 0.05). Dotted line, detection limit. Download [file mbo004162962sf2.tif]

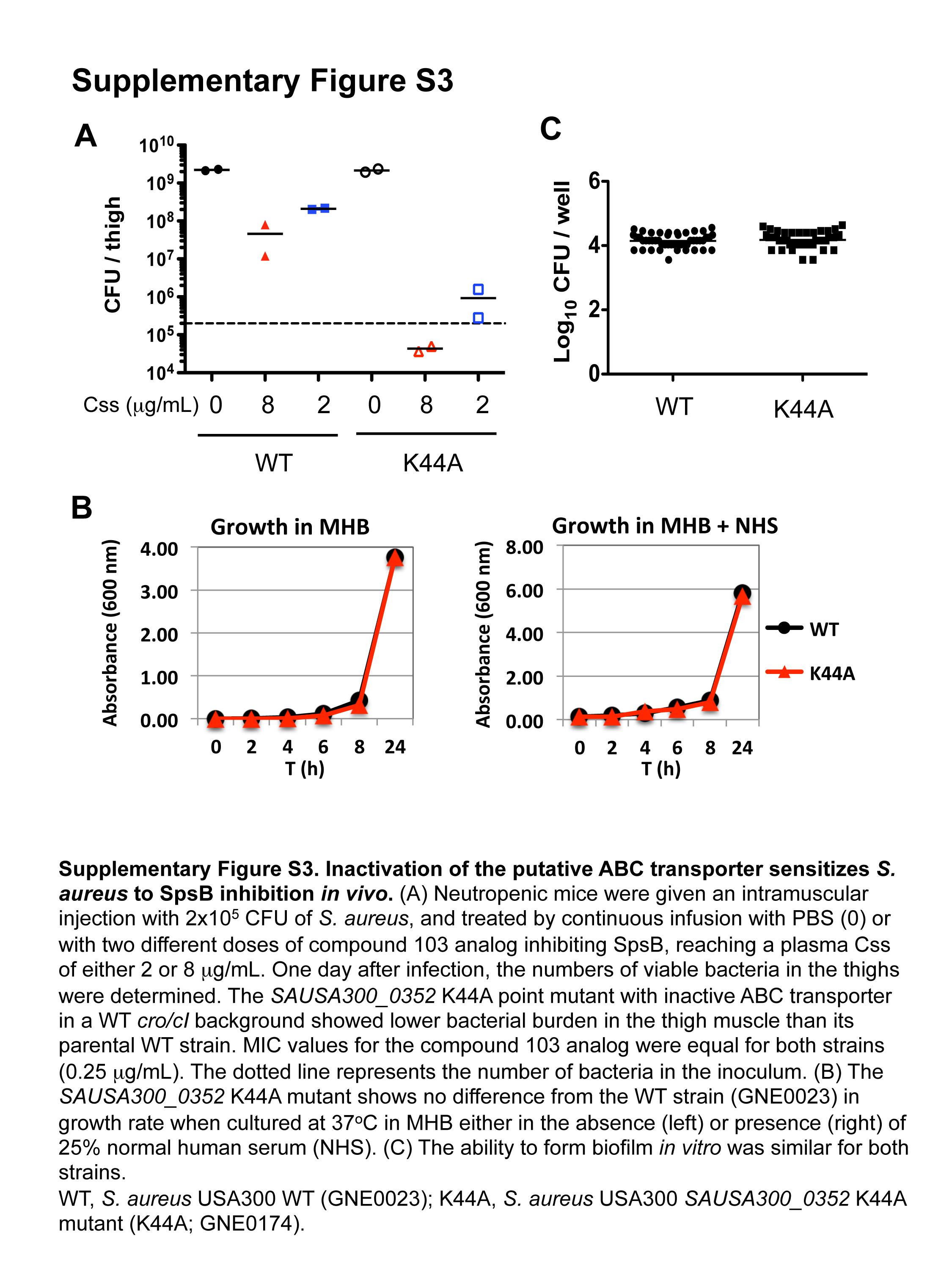

Supplement: Figure S3 — Inactivation of the putative ABC transporter sensitizes S. aureus to SpsB inhibition in vivo. (A) Neutropenic mice were given an intramuscular injection with 2 × 10^5^ CFU of S. aureus and treated by continuous infusion with PBS (0) or with two different doses of compound 103 analog, which inhibits SpsB, reaching a plasma concentration at steady state (Css) of either 2 or 8 µg/ml. One day after infection, the numbers of viable bacteria in the thighs were determined. The SAUSA300_0352(K44A) point mutant with inactive ABC transporter in a WT cro/cI background showed a lower bacterial burden in the thigh muscle than did its parental WT strain. The MICs of the compound 103 analog were equal for both strains (0.25 µg/ml). The dotted line represents the number of bacteria in the inoculum. (B) The SAUSA300_0352(K44A) mutant shows no difference from the WT strain (GNE0023) in growth rate when cultured at 37°C in MHB either in the absence (left) or presence (right) of 25% normal human serum (NHS). (C) The ability to form biofilm in vitro was similar for both strains. WT, S. aureus USA300 WT (strain GNE0023); K44A, S. aureus USA300 SAUSA300_0352(K44A) mutant (strain GNE0174). Download [file mbo004162962sf3.tif]

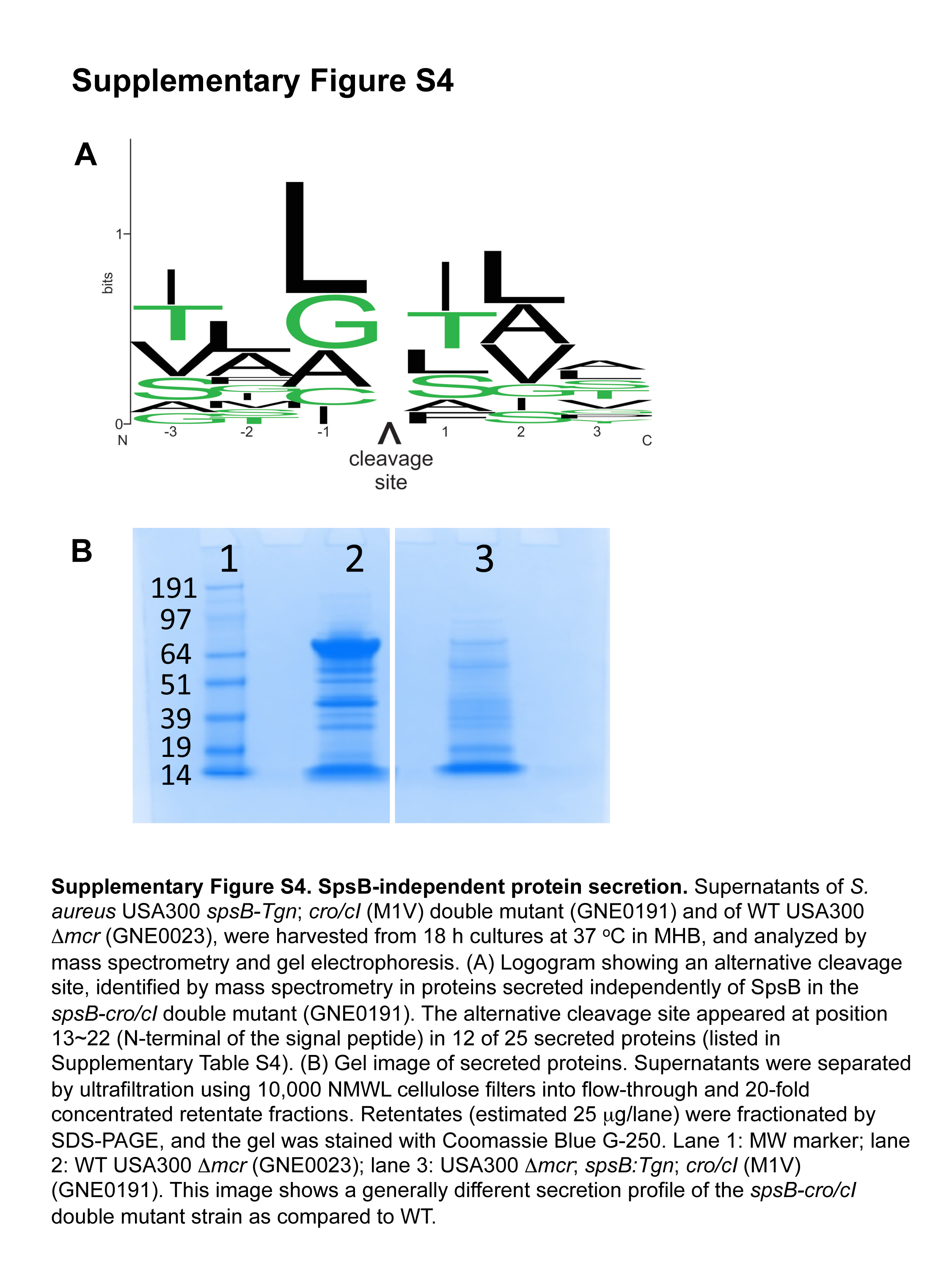

Supplement: Figure S4 — SpsB-independent protein secretion. Supernatants of S. aureus USA300 spsB::Tgn cro/cI(M1V) double mutant (strain GNE0191) and of WT USA300 Δmcr (strain GNE0023) were harvested from 18-h cultures grown at 37°C in MHB and analyzed by mass spectrometry and gel electrophoresis. (A) Logogram showing an alternative cleavage site identified by mass spectrometry in proteins secreted independently of SpsB in the spsB cro/cI double mutant (strain GNE0191). The alternative cleavage site appeared at various positions between 13 and 22 (N terminal to the signal peptide) in 12 of 25 secreted proteins (listed in Table S4 in the supplemental material). (B) Gel image of secreted proteins. Supernatants were separated by ultrafiltration using 10,000 nominal molecular weight limit (NMWL) cellulose filters into flowthrough and 20-fold-concentrated retentate fractions. Retentates (estimated to be 25 µg/lane) were fractionated by SDS-PAGE, and the gel was stained with Coomassie blue G-250. Lane 1, MW marker; lane 2, WT USA300 Δmcr (strain GNE0023); lane 3, USA300 Δmcr spsB::Tgn cro/cI(M1V) (strain GNE0191). This image shows that the secretion profile of the spsB cro/cI double mutant strain is generally different from that of the WT. Download [file mbo004162962sf4.tif]
