# Supplementary material for: A Putative Bacterial ABC Transporter Circumvents the Essentiality of Signal Peptidase
Source: mBio. 2016 Sep 6;7(5):e00412-16. doi: 10.1128/mBio.00412-16 (PMC5013292; doi:10.1128/mBio.00412-16)
Supplement: Table S1 — cro/cI nucleotide and amino acid changes leading to reduced sensitivity to compound 103. Shown are all mutations found inside or upstream of the SAUSA300_0350 (cro/cI) gene. Each of these mutations resulted in an increase in the MIC for compound 103 by at least 16-fold compared to the MIC for USA300 WT. [file mbo004162962st1.docx]

**Supplementary Table S1. c*ro/cI* nucleotide and amino acid changes leading to reduced sensitivity to compound 103.** Shown are all mutations found inside or upstream of the *SAUSA300_0350* (*cro/cI*) gene. Each of these mutations resulted in an increase in MIC for compound 103 by at least 16 fold as compared to USA300 WT.

| Nucleotide change in or upstream of the *SAUSA300_0350* (*cro/cI*) gene | Amino acid change in predicted Cro/cI protein |
| --- | --- |
| G3A | M1V |
| G26A | R9Q |
| C31T | R11 stop |
| C46T | Q16 stop |
| C52T | Q18 stop |
| G58A | A20T |
| C59T | A20V |
| C64T | Q22 stop |
| C68A | A23E |
| A81T | R27S |
| C86A | T29N |
| T91C | S31P |
| C92T | S31L |
| C92A | S31 stop |
| G102T | E34D |
| C118T | P40S |
| C122T | S41L |
| A157 insertion | N53K/E54 stop |
| G>T 14 bp upstream of *cro/cI* | n/a |
| G>A 62 bp upstream of *cro/cI* | n/a |
| CAAGCGGGCGTTT insertion after 63rd bp of *cro/cI* | R27S/Q28G/T29R/I30F/  S31K/L32T/I33N/E34H/  R35I/N36A/N37N/F38 stop |
