# Supplementary material for: A Putative Bacterial ABC Transporter Circumvents the Essentiality of Signal Peptidase
Source: mBio. 2016 Sep 6;7(5):e00412-16. doi: 10.1128/mBio.00412-16 (PMC5013292; doi:10.1128/mBio.00412-16)
Supplement: Table S2 — cro/cI(M1V) mutant of S. aureus USA300 exhibits unaltered sensitivity to antibiotics of various classes. [file mbo004162962st2.docx]

**Supplementary Table S2. *cro/cI* (M1V) mutant *S. aureus* USA300 exhibits unaltered sensitivity to antibiotics of various classes.**

| **Antibiotic** | **MIC of compound 103 (μg/mL)** | |
| --- | --- | --- |
|  | ***S. aureus* USA300 WT** | ***S. aureus* USA300 *cro/cI* (M1V) (GNE0117)** |
| Vancomycin | 2 | 1 |
| Linezolid | 4 | 4 |
| Oxacillin | 64 | 64 |
| Daptomycin | 2 | 2 |
| Rifampicin | 0.06 | 0.06 |
| Gentamycin | 1 | 1 |
| Clindamycin | 0.25 | 0.25 |
| Erythromycin | >64 | >64 |
| Gatifloxacin | 0.5 | 0.5 |
| Novobiocin | 0.25 | 0.25 |
