# Supplementary material for: A Putative Bacterial ABC Transporter Circumvents the Essentiality of Signal Peptidase
Source: mBio. 2016 Sep 6;7(5):e00412-16. doi: 10.1128/mBio.00412-16 (PMC5013292; doi:10.1128/mBio.00412-16)
Supplement: Table S3 — Genes that are overexpressed by at least twofold in the cro/cI(M1V) mutant of USA300 (strain GNE0117) compared to their expression in WT USA300 by RNA sequencing. The essential role of the clearly outstanding top four genes, which are organized in an operon with cro/cI itself, in resistance to compound 103 was confirmed experimentally by mutagenesis analysis (this study). For more details, see the text and Fig. 2A [file mbo004162962st3.docx]

**Supplementary Table S3**. **Genes that are overexpressed by at least 2 fold in *cro/cI*(M1V) mutant USA300 (GNE0117) as compared to WT USA300 by RNA sequencing.** The essential role of the clearly outstanding top four genes, which are organized in an operon with *cro/cI* itself, in resistance to compound 103 was confirmed experimentally by genetic mutagenesis analysis (this study). For more details, see the text and Figure 2A of the main manuscript.

| **Gene Symbol** | **Gene Name** | **USA300 NRS384 genome location start** | **USA300 NRS384 genome location end** | **Linear fold change** | **Adjusted P-value** |
| --- | --- | --- | --- | --- | --- |
| - | hypothetical protein (SAUSA300_0531; putative membrane protein) | 401758 | 402471 | 91.1 | 0.00E+00 |
| - | Cro/CI family transcriptional regulator-like (SAUSA300_0530; Cro/cI) | 401558 | 401761 | 88.6 | 9.28E-284 |
| - | ABC transporter ATP-binding protein (SAUSA300_0532; putative ATPase) | 402496 | 403338 | 78.2 | 0.00E+00 |
| - | hypothetical protein (SAUSA300_0533; putative permease) | 403338 | 403967 | 42.2 | 0.00E+00 |
| - | iron compound ABC transporter iron | 2309725 | 2310708 | 6.1 | 1.53E-25 |
| - | ABC transporter permease | 692432 | 693268 | 5.3 | 4.54E-17 |
| - | ABC transporter ATP-binding protein | 693262 | 694005 | 4.1 | 7.33E-10 |
| - | ABC transporter substrate-binding protein | 691506 | 692435 | 3.7 | 1.26E-10 |
| fhuA | ferrichrome transport ATP-binding protein fhuA | 708126 | 708923 | 3.6 | 2.67E-16 |
| - | iron compound ABC transporter permease | 2308682 | 2309713 | 2.8 | 7.33E-10 |
| ilvB | acetolactate synthase large subunit | 2166584 | 2168353 | 2.7 | 1.41E-04 |
| - | hypothetical protein | 326166 | 327689 | 2.7 | 7.92E-09 |
| leuS | rhodanese-like domain-containing protein | 1884789 | 1885100 | 2.6 | 1.33E-04 |
| - | Regulatory RNA | 690541 | 690980 | 2.6 | 1.97E-05 |
| fhuB | ferrichrome transport permease fhuB | 708959 | 709963 | 2.6 | 3.94E-15 |
| - | leucyl-tRNA synthetase | 1885122 | 1887539 | 2.5 | 6.32E-05 |
| ilvD | dihydroxy-acid dehydratase | 2164868 | 2166556 | 2.5 | 2.73E-04 |
| - | hypothetical protein | 2454328 | 2455587 | 2.4 | 2.01E-09 |
| purD | phosphoribosylamine--glycine ligase | 1069428 | 1070675 | 2.4 | 1.38E-05 |
| - | MarR family transcriptional regulator | 2507167 | 2507613 | 2.4 | 2.39E-06 |
| gltT | proton/sodium-glutamate symport protein | 2504945 | 2506222 | 2.4 | 3.55E-05 |
| gatB | aspartyl/glutamyl-tRNA amidotransferase subunit | 2041044 | 2042471 | 2.3 | 2.40E-11 |
| - | transporter gate domain-containing protein | 2306111 | 2307427 | 2.3 | 1.95E-04 |
| - | ABC transporter permease | 198050 | 198811 | 2.3 | 1.86E-04 |
| - | hypothetical protein | 1153841 | 1154191 | 2.3 | 2.24E-05 |
| - | hypothetical protein | 926492 | 926866 | 2.3 | 1.07E-03 |
| - | Na+/H+ antiporter family protein | 925157 | 926473 | 2.3 | 1.09E-03 |
| oppC | oligopeptide ABC transporter permease | 975069 | 976139 | 2.3 | 2.14E-09 |
| purH | bifunctional | 1067928 | 1069406 | 2.2 | 1.38E-04 |
| - | HemK family modification methylase | 2232829 | 2233581 | 2.2 | 3.29E-08 |
| - | ABC transporter ATP-binding protein | 2479029 | 2479694 | 2.2 | 2.69E-11 |
| - | putative helicase | 2614818 | 2617679 | 2.2 | 1.74E-07 |
| - | hypothetical protein | 755871 | 756323 | 2.2 | 1.70E-06 |
| - | fibrinogen-binding protein | 1150654 | 1150983 | 2.2 | 2.42E-10 |
| hisG | ATP phosphoribosyltransferase catalytic subunit | 2839076 | 2839690 | 2.2 | 7.10E-04 |
| - | hypothetical protein | 200072 | 200431 | 2.2 | 5.05E-13 |
| - | hypothetical protein | 198824 | 199855 | 2.2 | 2.10E-04 |
| hisD | histidinol dehydrogenase | 2837833 | 2839083 | 2.2 | 1.04E-03 |
| atpA | F0F1 ATP synthase subunit alpha | 2223267 | 2224775 | 2.2 | 4.80E-06 |
| efb | fibrinogen-binding protein | 1153190 | 1153687 | 2.2 | 1.59E-05 |
| atpG | F0F1 ATP synthase subunit gamma | 2222370 | 2223236 | 2.1 | 2.01E-05 |
| - | ABC transporter permease | 2479694 | 2480749 | 2.1 | 6.74E-07 |
| - | iron compound ABC transporter permease | 2307717 | 2308685 | 2.1 | 4.69E-05 |
| - | Regulatory RNA | 991511 | 991606 | 2.1 | 9.59E-05 |
| - | hypothetical protein | 2155242 | 2155466 | 2.1 | 2.94E-03 |
| purL | phosphoribosylformylglycinamidine synthase II | 1062671 | 1064860 | 2.1 | 7.52E-05 |
| - | hypothetical protein | 2458995 | 2460227 | 2.1 | 1.42E-08 |
| hfq | RNA chaperone host factor-1 protein | 1318604 | 1318837 | 2.1 | 2.33E-04 |
| - | hypothetical protein | 610145 | 610711 | 2.1 | 1.01E-03 |
| rplB | 50S ribosomal protein L2 | 2368559 | 2369392 | 2.1 | 3.08E-07 |
| - | hypothetical protein | 639461 | 639829 | 2.1 | 1.05E-06 |
| brnQ | branched-chain amino acid transport system II | 217161 | 218516 | 2.1 | 7.46E-04 |
| putP | high affinity proline permease | 2044612 | 2046150 | 2.1 | 2.89E-05 |
| - | homoserine dehydrogenase | 1343384 | 1344664 | 2.1 | 8.94E-05 |
| purF | amidophosphoribosyltransferase | 1064839 | 1066323 | 2.1 | 3.68E-04 |
| - | hypothetical protein | 2155526 | 2156605 | 2.1 | 5.17E-03 |
| hisC | histidinol-phosphate aminotransferase hisC | 2836804 | 2837817 | 2.1 | 6.19E-03 |
| fusA | elongation factor G | 594422 | 596503 | 2.0 | 6.20E-07 |
| - | hypothetical protein | 108303 | 108785 | 2.0 | 7.95E-06 |
| atpD | F0F1 ATP synthase subunit beta | 2220936 | 2222348 | 2.0 | 2.58E-05 |
| purN | phosphoribosylglycinamide formyltransferase | 1067347 | 1067913 | 2.0 | 6.99E-04 |
| - | putative lipoprotein | 197079 | 198053 | 2.0 | 3.08E-03 |
| pknB | protein kinase | 1218143 | 1220137 | 2.0 | 6.25E-05 |
