# Supplementary material for: A Putative Bacterial ABC Transporter Circumvents the Essentiality of Signal Peptidase
Source: mBio. 2016 Sep 6;7(5):e00412-16. doi: 10.1128/mBio.00412-16 (PMC5013292; doi:10.1128/mBio.00412-16)
Supplement: Table S4 — SpsB-independent processing of secreted proteins. Proteins secreted into the culture supernatants of S. aureus WT USA300 Δmcr (strain GNE0023) and USA300 Δmcr spsB::Tgn cro/cI(M1V) (strain GNE0191), cultured for 18 h at 37°C in MHB, were analyzed by mass spectrometry. Proteins detected in the supernatants of USA300 Δmcr spsB::Tgn cro/cI(M1V) were secreted independently of SpsB. SpsB cleavage sites in proteins secreted by WT USA300 Δmcr were confirmed either by the presence of a peptide whose N terminus matched the right flanking sequence in the trypsin digest of secreted proteins retained by a 10-kDa filter or by a peptide with the matching C-terminal sequence identified in the filter’s flowthrough. The observed N- or C-terminal flanking residues are indicated in boldface. For the first 12 proteins in this list, we predicted a consensus cleavage motif (see Fig. S4A in the supplemental material). [file mbo004162962st4.docx]

**Supplementary Table S4. SpsB-independent processing of secreted proteins.** Proteins secreted into the culture supernatants of *S. aureus* WT USA300 Δ*mcr* (GNE0023) and USA300 Δ*mcr*; *spsB:Tgn*; *cro/cI* (M1V) (GNE0191), cultured for 18 h at 37 ^o^C in MHB, were analyzed by mass spectrometry. Proteins detected in the supernatants of USA300 Δ*mcr*; *spsB:Tgn*; *cro/cI* (M1V) were secreted independently of SpsB. SpsB cleavage sites in proteins secreted by WT USA300 Δ*mcr* were confirmed either by the presence of a peptide with n-terminus matching the right flanking sequence in the trypsin digest of secreted proteins retained by a 10 kDa filter, or by a peptide with the matching c-terminal sequence identified in the filter’s flow-through. The observed n- or c-terminal flanking residues are indicated in bold. For the first 12 proteins in this list, we predicted a consensus cleavage motif (see Supplementary Figure S4A).

| **Reference** | **Protein name** | **SpsB-dependent cleavage detected in supernatant of WT USA300 Δ*mcr* (GNE0023)** | | | | | **SpsB-independent cleavage detected in supernatant of USA300 Δ*mcr*; *spsB:Tgn*; *cro/cI* (M1V) (GNE0191)** | | | |
| --- | --- | --- | --- | --- | --- | --- | --- | --- | --- | --- |
|  |  | **No. of peptide hits** | **Amino acid position** | | **Left flank** | **Right flank** | **No. of peptide hits** | **Amino acid position** | **Left flank** | **Right flank** |
| **Secreted in USA300 Δ*mcr*; *spsB:Tgn*; *cro/cI* (M1V) (GNE0191) with apparent alternative processing around residues 14 to 16 with new sequence specificity (n=12)** | | | | | | | | | | |
| ATL_STAAM\|Q931U5 | Bifunctional autolysin | 127 | 30 | **VQA** | | **AET** | 93 | 16 | VAL | **TLV** |
| CHIPS_STAAN\|Q99SU8 | Chemotaxis inhibitory protein | 5 | 29 | AKA | | **FTF** | 2 | 16 | SFL | **TAG** |
| HLGA_STAAM\|P0A071 | Gamma-hemolysin component A | 10 | 30 | SKA | | **ENK** | 6 | 16 | VGL | **IAP** |
| ISDC_STAAM\|Q99UX3 | Iron-regulated surface determinant protein C | 4 | 29 | ANA | | **ADS** | 5 | 19 | III | **IAT** |
| Q7A090_STAAW\|Q7A090 | MW2130 protein | 5 | 31 | **ASA** | | DSN | 7 | 10 | VTA | **TLA** |
| Q7A1B8_STAAW\|Q7A1B8 | Glycerophosphoryl diester phosphodiesterase | 27 | 31 | AGA | | **EQT** | 30 | 22 | TMG | **FLS** |
| Q7A1N8_STAAW\|Q7A1N8 | Uncharacterized protein | 24 | 28 | AEA | | **ASG** | 24 | 13 | IAC | **SVV** |
| Q8NWL8_STAAW\|Q8NWL8 | Panton-Valentine leukocidin chain S | 42 | 29 | **SKA** | | **DNN** | 14 | 15 | SLG | **IIT** |
| Q8NWL9_STAAW\|Q8NWL9 | Panton-Valentine leukocidin chain F | 35 | 25 | **VDA** | | **AQH** | 26 | 16 | IAL | **LLL** |
| Q8NX49_STAAW\|Q8NX49 | Alpha-Hemolysin | 61 | 27 | **ANA** | | ADS | 26 | 15 | TLL | **LGS** |
| Q8NYD4_STAAW\|Q8NYD4 | MW0284 protein | 3 | 32 | AFA | | **KSS** | 12 | 14 | ASL | **SVA** |
| SCIN_STAAM\|Q931M7 | Staphylococcal complement inhibitor | 3 | 32 | **AQA** | | **STS** | 5 | 14 | TLA | **IVL** |
| **Secreted in USA300 Δ*mcr*; *spsB:Tgn*; *cro/cI* (M1V) (GNE0191) without apparent identifiable processing (n=13)** | | | | | | | | | | |
| SSAA2_STAAM\|Q99RX4 | Staphylococcal secretory antigen ssaA2 | 12 | 28 | **AHA** | | **SEQ** | 8 | 131 | GLG | **ASY** |
| ISAA_STAAM\|P65645 | Probable transglycosylase IsaA | 63 | 30 | **AHA** | | AEV | 116 | 3 | MK | **KTIM** |
| Q7A060_STAAW\|Q7A060 | SsaA protein | 5 | 28 | **ADA** | | AEN | 10 | 4 | MKK | **LVT** |
| Q7A1T8_STAAW\|Q7A1T8 | Uncharacterized protein | 3 | 11 | VLS | | **MSA** | 25 | 5 | KLK | **SLA** |
| Q8NXE3_STAAW\|Q8NXE3 | MW0863 protein | 14 | 31 | **VSA** | | **AEK** | 56 | 5 | KLK | **SFI** |
| LIP1_STAAM\|P65288 | Lipase 1 | 111 | 35 | **AQA** | | AEK | 100 | 11 | SIR | **KFS** |
| LIP2_STAAM\|Q99WQ6 | Lipase 2 | 323 | 38 | **AQA** | | SEK | 147 | 14 | SIR | **KYS** |
| LTAS_STAAM\|Q99VQ4 | Lipoteichoic acid synthase | 55 | 218 | **ALA** | | SED | 20 | 233 | KQR | **QTE** |
| LUKL2_STAAM\|Q931I5 | Uncharacterized leukocidin-like protein 2 | 19 | 30 | **ANS** | | AHK | 4 | 134 | WLK | **YPS** |
| PLS_STAAM\|Q931E9 | Putative surface protein SAV2496/SAV2497 | on my calendar 23 | 51 | AEA | | **AEN** | 15 | 72 | EVK | **IEE** |
| PRSA_STAAM\|P60747 | Foldase protein PrsA | 20 | 26 | ASA | | **TDS** | 69 | 7 | INK | **LIV** |
| Q8NXR3_STAAW\|Q8NXR3 | MW0627 protein | 6 | 26 | AQA | | **STQ** | 6 | 106 | ASR | **YGV** |
| SPA_STAAM\|P0A015 | Immunoglobulin G-binding protein A | 126 | 37 | **ANA** | | **AQH** | 136 | 11 | SIR | **KLG** |
