# Supplementary material for: A Putative Bacterial ABC Transporter Circumvents the Essentiality of Signal Peptidase
Source: mBio. 2016 Sep 6;7(5):e00412-16. doi: 10.1128/mBio.00412-16 (PMC5013292; doi:10.1128/mBio.00412-16)
Supplement: Table S5 — Bacterial strains and plasmids. [file mbo004162962st5.doc]

**Supplementary Table S5. Bacterial strains and plasmids.**

| **Strain ID** | **Genotype** | | **Description** | **Source / reference** |
| --- | --- | --- | --- | --- |
| **USA300 strains** | | | | |
| USA300 | | Wild-type (WT) | Clinical isolate (NRS384) | NARSA/BEI |
| GNE0023 | | USA300 Δ*mcr* | USA300 with a deletion of *mcr* | [11] |
| GNE0028 | | USA300 SpsB-high | USA300 with vector pMK4 expressing *spsB* driven by the *sarA* promoter | This study |
| GNE0040 | | USA300 SpsB-low | USA300 with genomic incorporation of *spsB* under IPTG-sensitive promoter *spac* | This study |
| GNE0174 | | USA300 Δ*mcr;* *SAUSA300_0352* (K44A) | USA300 with a deletion of *mcr* and a K44A substitution in *SAUSA300_0352* (putative ABC transporter ATPase) | This study |
| GNE0163 | | USA300 + pMK4 (empty plasmid) | USA300 with empty vector pMK4 | This study |
| GNE0162 | | USA300 + pMK4 (p*sarA-SAUSA300_0351_0352_0353*) | USA300 with vector pMK4 expressing the putative ABC transporter genes *SAUSA300_0351*, *_0352*, and *_0353*, driven by the *sarA* promoter | This study |
| GNE0117 | | USA300 *cro/cI* (M1V) | USA300 with a M1V substitution in *cro/cI* (*SAUSA300_0350*) | This study |
| GNE0214 | | USA300 *cro/cI* (E34D) | USA300 with a E34D substitution in *cro/cI* (*SAUSA300_0350*) | This study |
| GNE0215 | | USA300 *cro/cI* (R11stop) | USA300 with a R11 to stop codon substitution in *cro/cI* (*SAUSA300_0350*) | This study |
| GNE0216 | | USA300 *cro/cI* (Q22stop) | USA300 with a Q22 to stop codon substitution in *cro/cI* (*SAUSA300_0350*) | This study |
| GNE0217 | | USA300 *cro/cI* (N53K/54stop) | USA300 with a N53K substitution followed by 54 stop codon insertion in *cro/cI* (*SAUSA300_0350*) | This study |
| GNE0218 | | USA300 *cro/cI* (Q28R) | USA300 with a Q28R substitution in *cro/cI* (*SAUSA300_0350*) | This study |
| GNE0219 | | USA300 *cro/cI* (T29N) | USA300 with a T29N substitution in *cro/cI* (*SAUSA300_0350*) | This study |
| GNE0107 | | USA300 *cro/cI* (M1V) + pMK4 (empty plasmid) | USA300 with an M1V substitution in *cro/cI* (*SAUSA300_0350*) and empty vector pMK4 | This study |
| GNE0111 | | USA300 *cro/cI* (M1V) + pMK4 (p*sarA*-*cro/cI*) | USA300 with a M1V substitution in *cro/cI* (*SAUSA300_0350*) and vector pMK4 expressing *cro/cI* driven by the *sarA* promoter | This study |
| GNE0109 | | USA300 *cro/cI* (M1V) + pMK4 (p*cro/cI*-*cro/cI*) | USA300 with a M1V substitution in *cro/cI* (*SAUSA300_0350*) and vector pMK4 expressing *cro/cI* driven by the promoter of *cro/cI* (i.e., 50 bp immediately upstream of *cro/cI*) | This study |
| GNE0119 | | USA300 *cro/cI* (-14GàT) | USA300 with a GàT bp substitution at 14 bp upstream of *cro/cI* (*SAUSA300_0350*) | This study |
| GNE0096 | | USA300 *cro/cI* (-14GàT) + pMK4 (empty plasmid) | USA300 with a GàT bp substitution at 14 bp upstream of *cro/cI* (*SAUSA300_0350*) and empty pMK4 vector | This study |
| GNE098 | | USA300 *cro/cI* (-14GàT) + pMK4 (p*sarA*-*cro/cI*) | USA300 with a GàT bp substitution at 14 bp upstream of *cro/cI* (*SAUSA300_0350*) and pMK4 vector expressing *cro/cI* driven by the *sarA* promoter | This study |
| GNE0097 | | USA300 *cro/cI* (-14GàT) + pMK4 (p*cro/cI*-*cro/cI*) | USA300 with a GàT bp substitution at 14 bp upstream of *cro/cI* (*SAUSA300_0350*) and pMK4 vector expressing *cro/cI* driven by the promoter of *cro/cI* (ie., 50 bp region upstream of *cro/cI*) | This study |
| GNE0118 | | USA300 *cro/cI* (-62GàA) | USA300 with a GàA bp substitution at 62 bp upstream of *cro/cI* (*SAUSA300_0350*) | This study |
| GNE0099 | | USA300 *cro/cI* (-62GàA) + pMK4 (empty plasmid) | USA300 with a GàA bp substitution at 62 bp upstream of *cro/cI* (*SAUSA300_0350*) and empty pMK4 vector | This study |
| GNE0100 | | USA300 *cro/cI* (-62GàA) + pMK4 (p*cro/cI*-*cro/cI*) | USA300 with a GàA bp substitution at 62 bp upstream of *cro/cI* (*SAUSA300_0350*) and pMK4 vector expressing *cro/cI* driven by the promoter of *cro/cI* (ie., 50 bp region upstream of *cro/cI*) | This study |
| GNE0173 | | USA300 Δ*mcr;* *cro/cI* (Q16stop) | USA300 with a deletion of *mcr* and a Q16 stop codon substitution in *cro/cI* (*SAUSA300_0350*) | This study |
| GNE0220 | | USA300 Δ*mcr;* *cro/cI* (Q16stop); Δ(*SAUSA300_0351_0352_0353*) | USA300 with a deletion of *mcr*, a Q16 stop codon substitution in *cro/cI* (*SAUSA300_0350*), and a deletion of *SAUSA300_0351_0352_0353* | This study |
| GNE0209 | | USA300 Δ*mcr;* *cro/cI* (Q16stop); Δ*SAUSA300_0351* | USA300 with a deletion of *mcr*, a Q16 stop codon substitution in *cro/cI* (*SAUSA300_0350*), and a deletion of *SAUSA300_0351* (putative ABC transporter membrane protein) | This study |
| GNE0175 | | USA300 Δ*mcr;* *cro/cI* (Q16stop); *SAUSA300_0352* (K44A) | USA300 with a deletion of *mcr*, a Q16 stop codon substitution in *cro/cI* (*SAUSA300_0350*), and a K44A substitution in *SAUSA300_0352* (putative ABC transporter ATPase) | This study |
| GNE0210 | | USA300 Δ*mcr;* *cro/cI* (Q16stop); Δ*SAUSA300_0353* | USA300 with a deletion of *mcr*, a Q16 stop codon substitution in *cro/cI* (*SAUSA300_0350*), and a deletion of *SAUSA300_0353* (putative ABC transporter permease) | This study |
| GNE0190 | | USA300 Δ*mcr;* *spsB:Tgn*; pNL9164-*ltrA* | USA300 with a deletion of *mcr*, a TargeTron insertion in *spsB*, and plasmid pNL9164-*ltrA* encoding LtrA | This study |
| GNE0191 | | USA300 Δ*mcr;* *spsB:Tgn*; *cro/cI*(M1V) | USA300 with a deletion of *mcr*; a TargeTron insertion in *spsB*; and a M1V substitution in *cro/cI* (*SAUSA300_0350*); derived from GNE0190 and lacking pNL9164-*ltrA* | This study |
|  | |  |  |  |
| **Other *S. aureus* strains** | | | | |
| NRS100 | COL | | Wild type (clinical isolate) | NARSA/BEI |
| NRS123 | USA400 | | Wild type (clinical isolate) | NARSA/BEI |
| NRS71 | USA200 | | Wild type (clinical isolate) | NARSA/BEI |
| NRS1 | Mu50 | | Wild type (clinical isolate) | NARSA/BEI |
| NRS384 | USA300 | | Wild type (clinical isolate) | NARSA/BEI |
| NRS106 | RN4220 | | Wild type | NARSA/BEI |
|  |  | |  |  |
| **Plasmids** |  | |  |  |
| pIMAY |  | | Targeting vector for transformation of NRS384 | [11] |
| pMK4 |  | | Expression vector for *S. aureus* | [19] |
| pNL9164(*ltrA*) |  | | Vector expressing LtrA which splices out a targetron insertion | Sigma |
|  |  | |  |  |
