# Supplementary material for: A Putative Bacterial ABC Transporter Circumvents the Essentiality of Signal Peptidase
Source: mBio. 2016 Sep 6;7(5):e00412-16. doi: 10.1128/mBio.00412-16 (PMC5013292; doi:10.1128/mBio.00412-16)
Supplement: Table S6 — Primers and probes used for qRT-PCR analysis of gene expression. Shown are the sequences of the forward and reverse primers and probes used to determine the expression of the corresponding genes, indicated in the left column, by qRT-PCR (see Fig. 3B). [file mbo004162962st6.docx]

**Supplementary Table S6. Primers and probes used for qRT-PCR analysis of gene expression.** Shown are the sequences of the forward and reverse primers and probes used to determine expression of the corresponding genes, indicated in the left column, by qRT-PCR (See Figure 3B)**.**

| **Gene** | **Genbank USA300** | **Forward primer** | **Reverse primer** | **Probe (FAM)** | **Fragment size** |
| --- | --- | --- | --- | --- | --- |
| *SAUSA300_0350 (cro/cI)* | 87160419 | GCTTAAACCAAACGCAACTTGCTA | CGCTCAATTAGCGATATGGTTTGTC | CAAGCGGGCGTTTCA | 67 |
| *SAUSA300_0351* | 87161964 | GTATTGAGAACTTGACGTTTGCGA | TGATTCATCAAAACAATGGCTTCTACCA | CGATTGTTGCGACGAT | 103 |
| *SAUSA300_0352* | 87161429 | AGGGATGCGAATGAAGATAGCTTT | CCATACCTGCAGTTGCTTCATCTAA | ACGATTGCGCTTTCTC | 86 |
| *SAUSA300_0353* | 87160824 | GGGCATACTCTATCATTTTCCCATTG | CCACGAAAGAAATAAGTAGTAGTATAGATGCA | TTGGCGCTGAAAACT | 102 |
| *rrsA* | 87159884:512890-514444 | GCCCCTTAGTGCTGCAGCTA | AGTTTCAACCTTGCGGTCGTA | ACCGATGCAACTAAGGAACCATACAATTCA | 67 |
